# A Simple Approximation to Bias in Gene–Environment Interaction Estimates When a Case Might Not Be the Case

**DOI:** 10.3389/fgene.2019.00886

**Published:** 2019-10-09

**Authors:** Iryna Lobach, Inyoung Kim, Alexander Alekseyenko, Siarhei Lobach, Li Zhang

**Affiliations:** ^1^Department of Epidemiology and Biostatistics, University of California, San Francisco, San Francisco, CA, United States; ^2^Department of Statistics, Virginia Tech University, Blacksburg, VA, United States; ^3^Department of Public Health Sciences, Medical University of South Carolina, Charleston, SC, United States; ^4^Applied Mathematics and Computer Science Department, Belarusian State University, Minsk, Belarus; ^5^Department of Medicine, University of California, San Francisco, San Francisco, CA, United States; ^6^Helen Diller Family Comprehensive Cancer Center, University of California, San Francisco, CA, United States

**Keywords:** Alzheimer’s disease, disease misclassification, bias, approximation, adaptive immune system

## Abstract

Case–control genetic association studies are often used to examine the role of the genetic basis in complex diseases, such as cancer and neurodegenerative diseases. The role of the genetic basis might vary by nongenetic (environmental) measures, what is traditionally defined as gene–environment interactions (G×E). A commonly overlooked complication is that the set of clinically diagnosed cases might be contaminated by a subset with a *nuisance* pathologic state that presents with the same symptoms as the pathologic state of interest. The genetic basis of the pathologic state of interest might differ from that of the nuisance pathologic state. Often, frequencies of the pathologically defined states within the clinically diagnosed set of cases vary by the environment. We derive a simple and general approximation to bias in G×E parameter estimates when the presence of the nuisance pathologic state is ignored. We then perform extensive simulation studies to show that ignoring the presence of the nuisance pathologic state can result in substantial bias in G×E estimates and that the approximation we derived is reasonably accurate in finite samples. We demonstrate the applicability of the proposed approximation in a study of Alzheimer’s disease.

## Introduction

Genetic association studies that estimate the relationship between gene–environment interactions (G×E) and a complex disease have the potential to provide valuable clues to the underlying etiology of complex diseases, such as cancer and neurodegenerative disease. Case–control studies sample a set of cases and a set of healthy controls conditionally on the disease status that is often defined based on the observed clinical diagnosis. A commonly overlooked complication is that multiple pathologic mechanisms might share symptoms and hence result in the same clinical diagnosis; i.e., the set of cases might be contaminated by a subset with a nuisance pathologic diagnosis. Frequencies of the pathologic diagnosis of interest within the set of clinically diagnosed cases might vary by the environment.

Our motivating study is a genome-wide association study (GWAS) of late-onset Alzheimer’s disease (AD), a neurodegenerative disorder that is clinically characterized by progressive mental decline but histopathologically defined by highly abundant amyloid deposits and neurofibrillary tangles in the brain (Potter and Wisniewski, 2012). Recent biomarker studies of AD (Salloway et al., 2014; Salloway and Sperling, 2015) reported that 36% of ApoE ε4 noncarriers and 6% of ApoE ε4 carriers clinically diagnosed with AD do not have evidence of amyloid deposition and hence do not qualify for the *pathologic (true)* diagnosis of AD.

We are interested in estimating the association between the genetic variants serving adaptive immune systems and the true, pathologically confirmed, AD status. The effect of the genetic variants might vary by the ApoE ε4 status, and in the context of this study, we define ApoE ε4 to be the environment. It is possible that the symptoms resulting in the clinical diagnosis are manifestations of an underlying polygenic mechanism, and hence, the clinical diagnosis is a surrogate of the *true* diagnosis. It is also possible that the symptoms with and without the amyloid deposition evidence represent diverse mechanisms each with a distinct genetic basis. In the latter case, the usual logistic model with the clinical diagnosis as an outcome is based on a misclassified disease status and hence misspecifies the link between G×E and the AD status.

Traditionally, case–control GWASs are analyzed in a logistic regression model as if the data are collected prospectively based on a justification provided by Prentice and Pyke (1979). In the situation when the disease status is misclassified with frequencies varying by the environment, the result of Prentice and Pyke (1979) does not naturally extend.

Extensive literature (Carroll et al., 2006; Buonaccorsi, 2010; Demidenko, 2013) reports on how the estimates of the main genetic effects can be biased in situations when the disease status is misclassified. We have recently examined bias in G×E when misclassification probabilities vary by the environment and proposed a solution that alleviates the bias (Lobach et al., 2018). This solution requires optimization of a complex nonlinear function. Interestingly, Neuhaus (1999) derived a general approximation to the bias in a univariate setting when the data are collected prospectively and are analyzed in the logistic regression model. We extend the literature by deriving a general theoretical bias and a convenient approximation to the bias for G×E when the data are collected retrospectively and hence in a logistic regression model where both the design of data collection and presence of the nuisance pathologic diagnosis in the set of cases are ignored. The approximation that we have derived does not require optimization of a complex nonlinear function and provides a convenient understanding about the magnitude and directionality of the bias.

Our paper proceeds as follows. We first describe the setting and the approximation that we have derived in the *Results* section. We next describe a series of simulation experiments showing that the approximation that we have derived is accurate relative to the empirical estimates and apply the approximation to the study of AD. We conclude the paper by a brief discussion.

## Results

We define *G* as the genotype, e.g. single-nucleotide polymorphisms (SNPs) measured at multiple locations. Let *X* be the environmental variable that interacts with *G*. For clarity of presentation, we base the development on binary variables, *G* and *X*. We assume that the genotype is independent of all environmental variables and the genotypes follow the Hardy–Weinberg equilibrium: *G* ∼ *Q*(*g*, θ).

If θ is the frequency of minor allele *a* when the major allele is A, then the Hardy–Weinberg equilibrium model (Hardy, 1908) states

$$G\sim Q\left(g,\theta \right)=Pr\left(G=g\text{|}\theta \right)=\{\begin{array}{c} 2\times \theta \times \left(1-\theta \right),\;if\;g=Aa\\ \;\;\;\;\;\;\theta^{2},\;\;\;\;\;\;\;\;\;\;\;\;\;\;if\;g=aa\\ {\left(1-\theta \right)}^{2},\;\;\;\;\;\;if\;g=AA \end{array}.$$

We define *D*^CL^ = {0, 1} as the observed clinical disease status defined based on a set of symptoms. Suppose that the same set of symptoms can be caused by two distinct pathophysiologic mechanisms. Let *D* be the *true* disease status defined based on the underlying pathology, where *D* = 1 indicates the disease of interest, while *D* = 1^*^ is the nuisance disease. Thus, pr(*D* = 1) + pr(*D* = 0) + pr(*D* = 1^*^) = 1. For ethical and/or budgetary reasons, it might not be possible to measure the underlying pathology on all patients; hence, *D* is latent.

In the setting of AD, *D* = 1 indicates the true AD state, i.e. the clinical diagnosis and evidence of amyloid deposition, and *D* = 1^*^ indicates the nuisance disease state, e.g. vascular dementia, Lewy body disease, and hippocampal sclerosis (Salloway and Sperling, 2015), clinically diagnosed as AD. In the setting of breast cancer, for example, the disease state of interest might be an estrogen receptor (ER)+ breast cancer, while the nuisance disease state is an ER− cancer. A hypothesis then could be that a set of the genetic variants is associated with the ER+ disease state, but not the ER− disease state.

Instead, an evaluation might be performed on a subset of patients or in an external reliability study. We define τ(*X*) = pr(*D* = 1|*D*^CL^ = 1, *X*) to be the frequency of the *true* diagnosis of interest within the clinically diagnosed set that varies by the environmental variable *X*. In our setting, the clinical diagnosis of healthy controls corresponds to *D* = 0; hence, pr(*D* = 0|*D*^CL^ = 0, *X*) = 1, and pr(*D* = 1|*D*^CL^ = 0, *X*) = 0. We let probabilities of the clinical and *true* diagnoses in the population be $\pi_{d^{\text{cl}}}=\text{pr}\left(D^{\text{CL}}=d^{\text{cl}}\right)$ and π*_d_* = pr(*D* = *d*), respectively. Let 𝒮(*X*) = pr(*D* = 0|*D*^CL^ = 1, *X*). For clarity of presentation, we suppose that all variables are binary. The setting can be easily extended to accommodate multilevel categorical variables.

We first consider a binary setting where the risk parameters are defined in terms of *D* = 1 vs. *D* = 1^*^ and *D* = 0 combined. Then, the true risk model in terms of coefficients *B* = (β_0_, β*_G_*, β*_X_*, β*_G_*_×_*_X_*) is

(1) $$\begin{array}{l} \text{log}\left\{\frac{\text{pr}\left(D=1\;\text{|}G=g,X=x\right)}{\text{pr}\left(D=\;1^{*}\;or\;0\;\text{|}G=g,X=x\right)}\right\}=\\ \beta_{0}+\beta_{G}\times g+\beta_{X}\times x+\beta_{G\times X}\times g\times x. \end{array}$$

In the second setting, we consider that the risk model is defined separately for *D* = 1 vs. *D* = 0 in terms of *B* = (껐β_0_, β*_G_*, β*_X_*, β*_G_*_×_*_X_*) and for *D* = 1^*^ vs. *D* = 0 in terms of ${Β}^{*}=\left(\beta_{0}^{*},\beta_{G}^{*},\beta_{X}^{*},\beta_{G\times X}^{*}\right)$ by

(2) $$\begin{array}{l} \begin{array}{l} \text{log}\left\{\frac{{\text{pr}}_{B,B^{*}}\left(D=1\;\text{|}G=g,X=x\right)}{{\text{pr}}_{B,B^{*}}\left(D=\;0\;\text{|}G=g,X=x\right)}\right\}=\\ \beta_{0}+\beta_{G}\times g+\beta_{\text{X}}\times x+\beta_{G\times X}\times g\times x; \end{array}\\ \begin{array}{l} \text{log}\left\{\frac{{\text{pr}}_{B,B^{*}}\left(D=1^{*}\text{|}G=g,X=x\right)}{{\text{pr}}_{B,B^{*}}\left(D=\;0\text{|}G=g,X=x\right)}\right\}=\\ \beta_{0}^{*}+\beta_{0}^{*}\times g+\beta_{X}^{*}\times x+\beta_{G\times X}^{*}\times g\times x. \end{array} \end{array}$$

In equation (2), *B* and B^*^ might share coefficients, e.g. if $\beta_{X}=\beta_{X}^{*}$. While we define the parameters of interest to be *B* = (β_0_, β*_G_*, β*_X_*, β*_G_*_×_*_X_*), these parameters are independent and are different in models (1) and (2).

Suppose that the clinical–pathological diagnosis relationship is ignored and the clinical diagnosis is used as the outcome variable. Then, the risk model is

(3) $$\begin{array}{l} \text{log}\left\{\frac{{\text{pr}}_{\Gamma }\left(D^{\text{CL}}=1\text{|}G,X\right)}{{\text{pr}}_{\Gamma }\left(D^{\text{CL}}=0\text{|}G,X\right)}\right\}=\\ \gamma_{0}+\gamma_{G}\times g+\gamma_{X}\times x+\gamma_{G\times X}\times g\times x. \end{array}$$

Derivations provided in the Online (Kullback, 1959) Methods show that

(4) $$\gamma_{0}\approx \log\left\{\frac{1+S\left(0\right)}{1-S\left(0\right)}\right\}+\frac{1}{1+S\left(0\right)}\times \beta_{0};$$

(5) $$\begin{array}{l} \gamma_{\text{X}}\approx \log\left\{\frac{S\left(1\right)+e^{\beta_{0}+\beta_{X}}}{1-S\left(1\right)}\right\}-\log\left\{\frac{S\left(0\right)+e^{\beta_{0}}}{1-S\left(0\right)}\right\}+\\ \frac{e^{\beta_{0}}}{e^{\beta_{0}}+S\left(1\right)}\times \beta_{X}; \end{array}$$

(6) $$\gamma_{\text{G}}\approx \frac{e^{\beta_{0}}}{e^{\beta_{0}}+S\left(0\right)}\times \beta_{G};$$

(7) $$\begin{array}{l} \gamma_{G\times X}\approx \log\left[\frac{S(1)\times e^{\beta_{0}+\beta_{X}+\beta_{G}}}{1+\left\{1-S(1)\right\}\times e^{\beta_{0}+\beta_{X}+\beta_{G}}}\right]-\\ \log\left[\frac{S(0)\times e^{\beta_{0}+\beta_{G}}}{1+\left\{1-S(0)\right\}\times e^{\beta_{0}+\beta_{G}}}\right]-\log\left[\frac{S(1)\times e^{\beta_{0}+\beta_{X}}}{1+\left\{1-S(1)\right\}\times e^{\beta_{0}+\beta_{X}}}\right]+\\ \log\left[\frac{S(0)\times e^{\beta_{0}}}{1+\left\{1-S(0)\right\}\times e^{\beta_{0}}}\right]+\frac{1}{1+\left\{1-S(1)\right\}\times e^{\beta_{0}+\beta_{X}+\beta_{G}}}\times \beta_{G\times X}. \end{array}$$

### Remarks on Formulas (4)–(7)

1. The Online Methods section provides formulas (A11)–(A15) for the setting with environmental variable *Z* that does not interact with the genetics.
2. When the clinical diagnosis and pathologic disease status correspond, i.e. 𝒮(*X*) = 0 for *X* = 1 and *X* = 0, then all parameter estimates are unbiased.
3. If βG = 0, then γG = 0. Hence, the usual logistic regression yields the correct estimate of the null βG.
4. βG = 0 does not necessary result in γ0 = 0. Similarly, if βX = 0, then γX might not be zero, and if βG_×_X = 0, then γG_×_X might not be zero. Hence, the usual logistic regression does not yield the correct estimate of the null effect β0, βX, βG, and βG_×_X.
5. If βG = 0 and βG_×_X = 0, then γG = 0 and γG_×_X = 0. Hence, the usual logistic regression yields the correct estimate of the null βG and βG_×_X.
6. If the misclassification is nondifferential, i.e. 𝒮(0) = 𝒮(1), then if βX = 0, then γX = 0. That is, the usual logistic regression model yields the correct estimate of the null effect βX.
7. If the misclassification is nondifferential, i.e. 𝒮(0) = 𝒮(1), then if β0 = 0, βX = 0, βG = 0, and βG_×_X = 0, then γG_×_X = 0. That is, the usual logistic regression model yields the correct estimate of the null effect of βG_×_X.

We next suppose that the true model is (2), while the parameters are estimated based on a misspecified model (3).

Derivations provided in the Appendix show that

(8) $$\gamma_{0}\approx \log\left(e^{\beta_{0}}+e^{\beta_{0}^{*}}\right)\approx \beta_{0}^{*}+\frac{1}{1+e^{\beta_{0}^{*}}}\times \beta_{0};$$

(9) $$\begin{array}{l} \gamma_{G}\approx \log\left(e^{\beta_{0}+\beta_{G}}+e^{\beta_{0}^{*}+\beta_{G}^{*}}\right)-\\ \log\left(e^{\beta_{0}}+e^{\beta_{0}^{*}}\right)\approx \frac{e^{\beta_{0}}}{e^{\beta_{0}}+e^{\beta_{0}^{*}+\beta_{G}^{*}}}\times \beta_{G}; \end{array}$$

(10) $$\begin{array}{l} \gamma_{X}=\log\left(e^{\beta_{0}+\beta_{X}}+e^{\beta_{0}^{*}+\beta_{X}^{*}}\right)-\\ \log\left(e^{\beta_{0}}+e^{\beta_{0}^{*}}\right)\approx \frac{e^{\beta_{0}}}{e^{\beta_{0}}+e^{\beta_{0}^{*}+\beta_{X}^{*}}}\times \beta_{X}; \end{array}$$

(11) $$\begin{array}{l} \gamma_{G\times X}=\log\left(e^{\beta_{0}+\beta_{G}+\beta_{X}+\beta_{G\times X}}+e^{\beta_{0}^{*}+\beta_{G}^{*}+\beta_{X}^{*}+\beta_{G\times X}^{*}}\right)\\ -\log\left(e^{\beta_{0}+\beta_{X}}+e^{\beta_{0}^{*}+\beta_{X}^{*}}\right)-\log\left(e^{\beta_{0}+\beta_{G}}+e^{\beta_{0}^{*}+\beta_{G}^{*}}\right)+\\ \log\left(e^{\beta_{0}}+e^{\beta_{0}^{*}}\right)\approx \log\left(e^{\beta_{0}+\beta_{G}+\beta_{X}}+e^{\beta_{0}^{*}+\beta_{G}^{*}+\beta_{X}^{*}+\beta_{G\times X}^{*}}\right)-\\ \log\left(e^{\beta_{0}+\beta_{X}}+e^{\beta_{0}^{*}+\beta_{X}^{*}}\right)-\log\left(e^{\beta_{0}+\beta_{G}}+e^{\beta_{0}^{*}+\beta_{G}^{*}}\right)+\\ \log\left(e^{\beta_{0}}+e^{\beta_{0}^{*}}\right)+\frac{e^{\beta_{0}+\beta_{G}+\beta_{X}}}{e^{\beta_{0}+\beta_{G}+\beta_{X}}+e^{\beta_{0}^{*}+\beta_{G}^{*}+\beta_{X}^{*}+\beta_{G\times X}^{*}}}\times \beta_{G\times X}. \end{array}$$

### Remarks on Formulas (8)–(11):

1. The Online Methods section provides formulae (A19)–(A23) for the setting with environmental variable *Z* that does not interact with the genetics.
2. If β0 = 0 and $\beta_{0}^{*}=0$, then γ0 = 0. That is, the usual logistic regression yields the correct estimate of the null β0.
3. If βG = 0 and $\beta_{G}^{*}=0$, then γG = 0. Hence, in this case, the usual logistic regression yields the correct estimate of the null βG.
4. If βX = 0 and $\beta_{X}^{*}=0$, then γX = 0. Hence, in this case, the usual logistic regression yields the correct estimate of the null βX.
5. If βG = 0 and βG_×_X = 0, then $\beta_{G}^{*}=0$ and $\beta_{G\times X}^{*}=0$. Hence, the usual logistic regression yields the correct estimate of the null βG and βG_×_X.
6. If βG = 0, $\beta_{G}^{*}=0$, βX = 0, $\beta_{X}^{*}=0$, βG_×_X = 0, and $\beta_{G\times X}^{*}=0$, then γG_×_X = 0. Hence, if all these parameters are zero, the usual logistic regression yields the correct estimate of the null βG_×_X.

### Simulation Experiments

We perform a series of simulation experiments to evaluate the accuracy of the proposed approximation. We set our other parameters to be similar to the values observed in our GWAS of AD. We let the genotype (*G*) be a Bernoulli random variable with a frequency of 0.10 to mimic a SNP and allow its effect to follow a recessive or dominant model. We simulated age (*Z*_1_) to be Bernoulli with a frequency of 0.50, e.g. corresponding to a median split. Sex (*Z*_2_) is Bernoulli with pr(*Z*_2_ = 1) = 0.52. The binary variable *X* = {ε4+, ε4−}, which represents the ApoE ε4 status according to the presence or absence of the ε4 allele that occurs in approximately 14% of the population. Similarly to Salloway et al. (2014), we define the proportion of the nuisance disease within the clinical diagnosis as pr(*D* = 1^*^|*D*^CL^ = 1, ε4−) = 0.36 and pr(*D* = 1^*^|*D*^CL^ = 1, ε4+) = 0.06.

#### Setting A

We first simulate data according to model (1) while we estimate parameters in model (3) where the clinically diagnosed status is the outcome variable. The disease status is simulated according to model (1) with β*_G_*_×ε4_ varying from log(1) to log(8), and we set $\beta_{Z_{1}}$ to be 0, 0.5, 1, or 1.5. The other risk coefficients are β_0_ = −1, β*_G_* = −0.41, $\beta_{Z_{2}}=-0.08$, and β_ε4_ = log(8) = 2.1. We simulated 500 datasets with 3,000 cases and 3,000 controls. Shown in **Table S1** are parameter estimates when β*_G_*_×ε4_ = 0, obtained as an average (empirical estimate) and standard deviation (SD) across the 500 simulated datasets, theoretical values, and the simple approximation derived in (A11)–(A15). For all parameters, the empirical values are close to the approximation, with the average difference across all parameters being 0.11. The approximation was furthest away from the empirical estimate of the main effect of ApoE ε4 status, with the empirical estimate being 1.80, while the approximation is 1.30.

Shown in **Figures S1A–S1D** are the estimates [empirical estimate that is the average across 500 simulated datasets (AVE), theoretical estimate (TH) (A11)–(A15), and approximation (APX) (A11.1)–(A15.1)] across the levels of $\beta_{Z_{2}}$ that are indicated by color and across values of β*_G_*_×ε4_ across the panels of each graph. In all of these settings, the theoretical values and the approximations are accurate relative to the empirical estimates. The furthest from the empirical estimates are approximation for the main effect of the ApoE ε4 status with the difference mainly driven by the approximation.

To examine robustness of the theoretically derived magnitude of bias to misspecification of proportions of the nuisance disease, we calculated the theoretical values while underestimating the frequencies to be pr(*D* = 1^*^|*D*^CL^ = 1, ε4−) = 0.30 and pr(*D* = 1^*^|*D*^CL^ = 1, ε4+) = 0 (**Figures S2A–S2C** for γ*_G_*, γ_ε4_, and γ*_G_*_×ε4_, respectively). The approximation to bias is robust to this misspecification while overestimating the frequencies to be pr(*D* = 1^*^|*D*^CL^ = 1, ε4−) = 0.42 and pr(*D* = 1^*^|*D*^CL^ = 1, ε4+) = 0.12 (**Figures S3A–S3C** for γ*_G_*, γ_ε4_, and γ*_G_*_×ε4_, respectively).

#### Setting B

We next simulate the data according to risk model (2) while estimating parameters based on model (3). We simulate the disease status *D* = 1 vs. *D* = 0 based on parameters β_0_ = −1, β*_G_* = −0.69, $\beta_{Z_{1}}=0.10$, $\beta_{Z_{2}}=-0.083$, β_ε4_ = 1.3, and β*_G_*_×ε4_ = 1.099; and we simulate *D* = 1^*^ vs. *D* = 0 using $\beta_{0}^{*}=-1.7$,$\beta_{G}^{*}=0$, $\beta_{\varepsilon 4}^{*}=0.5$, and $\beta_{G\times \varepsilon 4}^{*}=0$ with main effects of *Z*_1_ and *Z*_2_ that are the same as those for *D* = 1 vs. *D* = 0. With these parameters, the frequencies of the disease of interest and the nuisance disease are pr(*D* = 1) = 5.1%, pr(*D* = 1^*^) = 12.5%, pr(*D* = 1|ε4+) = 45.4%, pr(*D* = 1^*^|ε4+) = 16.1%, pr(*D* = 1|ε4−) = 20%, and pr(*D* = 1^*^|ε4−) = 16.1%. **Table S2** (*n*_0_ = *n*_1_ = 3,000) presents empirical estimates, theoretical values (A16–A22), and approximations (A16.1–A22.1).

We first note that when the presence of the nuisance disease is ignored, estimates of β_0_, β_ε4_, β*_G_*_×ε4_, and β*_G_* are substantially biased. The approximation that we derived is accurate relative to the empirical averages of the parameter estimates. For example, the empirical estimate of γ_ε4_ is 1.08, while the approximation is 1.08. The empirical estimate of γ*_G_*_×ε4_ is −0.10, while the approximation is −0.05.

Shown in **Figures S4A, S4B** are the estimates [empirical estimate that is the average across 500 simulated datasets (AVE) and approximation (A20)–(A23) across values of $\beta_{G\times X}^{*}$ along the *x*-axis when β*_G_*_×ε4_ = 0 (**Figure S4A**) and when β*_G_*_×ε4_ = −0.9 (**Figures S4B**). In all of these settings, the theoretical values and the approximations are accurate relative to the empirical estimates.

#### Setting C

We next simulate the data with a smaller sample size and unequal number of cases and controls, i.e. 1,000 controls and 2,000 cases, with the rest of the setting being the same as Setting B. **Table S3** presents empirical estimates, theoretical values (A16–A22), and approximation (A16.1–A22.1). We note here that when the nuisance disease is ignored, the estimates of the coefficients are substantially biased. The approximation that we derived is reasonably accurate relative to the empirical averages of the estimates.

#### Setting D

We next performed a study where we misspecified rates of the nuisance disease state within the clinical diagnosis by 5%, with the rest of the setting being the same as Setting C. We considered three settings: 1) when the rate is overestimated by 5% for both ε4 carriers and ε4 noncarriers; 2) when the rate is underestimated by 5% for both ε4 carriers and ε4 noncarriers; and 3) when the rate is overestimated by 5% in ε4 carriers and overestimated by 5% in ε4 noncarriers. In settings 1 and 2, the rate is equally misspecified between cases and controls; hence, the degree to which the rate is differential, i.e. varies by ε4 status, is preserved. In setting 3, however, we also increased the degree to which the rate is differential, which would likely have more impact. In settings 1 and 2, we observed no impact on the estimates, as shown in **Table S4**. In setting 3, however, the estimates changed slightly. Hence, the estimates are more robust to misspecification that does not increase the degree to which the rate is differential.

### Analyses of Genetic Variants Serving Adaptive Immune System in AD

We applied the proposed analyses to a dataset collected as part of the Alzheimer’s Disease Genetics Consortium. The data consist of 1,245 controls and 2,785 cases. The average age (SD) of cases and controls are 72.1 (9.1) and 70.9 (8.8) years, respectively. Among cases, 1,458 (52.4%) are men; among controls, 678 (63.9%) are men. At least one ApoE ε4 allele is present in (64.5%) of cases and 365 (29.1%) of controls.

Illumina Human 660K markers have been mapped onto human chromosomes using the National Center for Biotechnology Information (NCBI) dbSNP database (https://www.ncbi.nlm.nih.gov/projects/SNP/). Chromosome location, proximal gene or genes, and gene structure location [e.g. intron, exon, intergenic, and untranslated region (UTR)] have been recorded for all SNPs. We inferred the adaptive immune system pathways based on the information from the Kyoto Encyclopedia of Genes and Genomes (www.genome.jp/kegg) (KEGG), Gene Ontology (GO) Consortium (www.geneontology.org), and Ariadne Genomics (www.ariadnegenomics.com). From these data with quality control measures (observed frequency of minor allele >5%), we inferred 133 SNPs to reside in genes serving the adaptive immune system.

It is of interest to examine a relationship between the pathologic diagnosis and each of the 133 SNPs (*G*), ApoE ε4 status (*X*), age (*Z*_1_), and sex (*Z*_2_). The effect of SNPs might vary by ApoE ε4; hence, we included the interaction between the genotype and ApoE ε4 status. The genetic variables are modeled using a binary indicator of the presence or absence of a minor allele.

We estimate parameters using the standard logistic model (3) that uses the clinical diagnosis as a surrogate of the pathophysiology. We assume that the proportion of nuisance disease is as estimated by the Salloway, S., and Sperling, R. (2015) study; i.e. the proportion of the nuisance disease within the clinically diagnosed set of cases is 36% in ApoE ε4 noncarriers and 6% in ApoE ε4 carriers. We first assume that the true model is (1); i.e. the susceptibility model is defined for amyloid-related AD symptoms vs. healthy controls and nonamyloid AD symptoms combined. In this setting, we estimate the magnitude of bias using approximation (A11.1–A15.1). We next assume that the true model is (2); i.e. the susceptibility model is defined for amyloid-related AD symptoms vs. controls and for non-amyloid-related symptoms vs. controls. We then estimate the magnitude of bias using (A19.1)–(A25.1).

**Figure 1:**
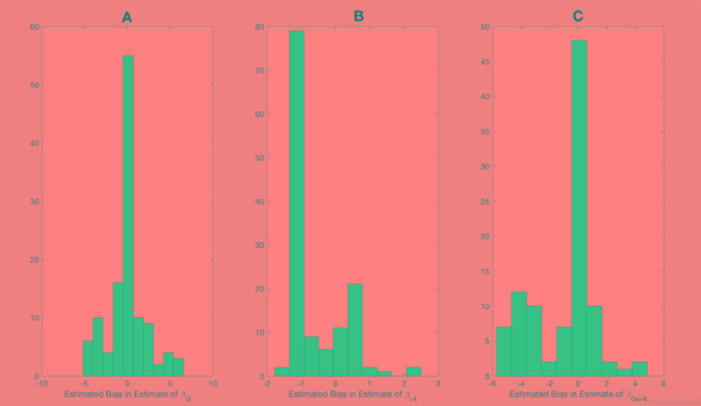
Bias in parameter estimates in Alzheimer's disease study assuming that the true model is (1) while parameters are estimated using model (3). Magnitude of bias is approximated using (A11.1)-(A15.1). **(A)** approximation to the bias in the main effect of genotype estimate, **(B)** approximation to the bias in the main effect of ApoE allele estimate, **(C)** approximation to the bias in the interaction between the genotype and ApoE allele estimate.

**Figure 2:**
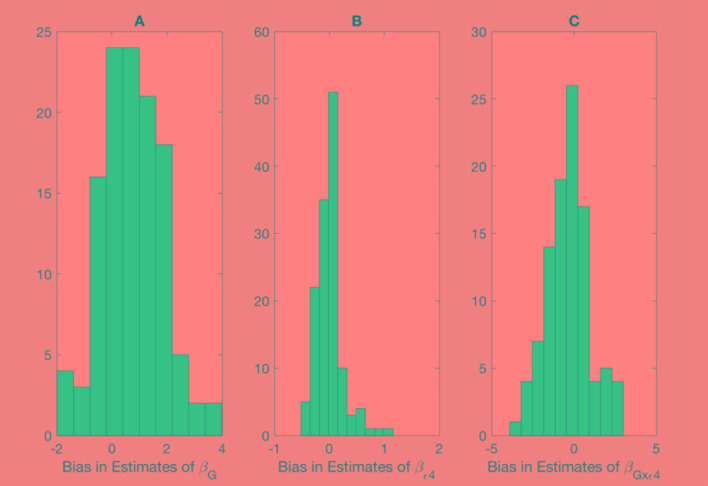
Bias in parameter estimates in Alzheimer's disease study assuming that the true model is (2) while parameters are estimated using model (3). Magnitude of bias is approximated using (A19.1)-(A23.1). **(A)** approximation to the bias in the main effect of genotype estimate, **(B)** approximation to the bias in the main effect of ApoE allele estimate, **(C)** approximation to the bias in the interaction between the genotype and allele estimate.

Shown on **Figures 1** and **2** are the estimated biases in the main effect of each SNP, ApoE ε4, and interaction between the SNPs and ApoE ε4 status. We first note that the bias in the estimate can be substantial. For example, in the usual logistic regression with the clinical diagnosis as an outcome variable, the main effect of rs597587 is statistically significant with Bonferroni correction (*p* < 0.05/133). The estimate is ${\hat{\gamma }}_{G}=-1.1$, while if model (1) is the true model, the bias is approximated to be 2.9 and the interactive effect is estimated to be ${\hat{\gamma }}_{G\times \varepsilon 4}=2.0$, *p* < 0.008, which means if the model was incorrectly specified, both main and interactive effects would be mistakenly interpreted. For another SNP, rs12111032, the main effect estimate is ${\hat{\gamma }}_{G}=0.78$ with *p* < 0.05/133, while the bias is −0.04 if model (1) is the true model and −0.17 if model (2) is the true model.

**Table 1** presents the estimates of main effects of SNPs and *p*-values obtained using the usual logistic regression model with the clinically diagnosed disease status as an outcome variable for SNPs with a *p*-value <0.05 for ${\hat{\gamma }}_{G}$ or ${\hat{\gamma }}_{G\times \varepsilon 4}$. We also mapped the genes from **Table 1** to the amyloid pathway using KEGG and GO. A SNP, rs10059242, residing in an intergenic region between HTR4 and ADRB2 (ADRB2 is found to be in the amyloid pathway) has the main effect of ${\hat{\gamma }}_{G}=1.5$ (*p* = 0.04) with bias almost equal to −0.9 (1) and 1.8 (2) and the G×E interaction of ${\hat{\gamma }}_{G\times \varepsilon 4}=-2.0$, (*p* = 0.04) with bias almost equal to −0.9 (1) and −2 (2).

**Table 1:** Estimate of the main effect of single-nucleotide polymorphisms (SNPs) $\hat{\left(\gamma_{G}\right)}$ and their interaction effect with ApoE ε4 status in the Alzheimer disease study.

SNP	Gene/intergenic region	Bias in ${\hat{\gamma }}_{G}$		${\hat{\gamma }}_{G}$ from model (3)		Bias in ${\hat{\gamma }}_{G\times \varepsilon 4}$		${\hat{\gamma }}_{G\times \varepsilon 4}$ from model (3)	
		(1) is the true model	(2) is the true model	Estimate	*p*-value	(1) is the true model	(2) is the true model	Estimate	*p*-value
rs401904	CD1D | CD1A	0.08	−0.05	−0.08	0.64	−0.08	0.25	0.83	0.04
rs1748383	N4BP2 | RHOH	0.07	−0.39	−0.07	0.69	−0.07	−0.68	0.82	0.04
rs13386118	CXCR4 | THSD7B	3.9	1.4	−3.6	0.003	2.9	−1.2	0	0.99
rs12692222	LOC100419686 | LOC151171	0.04	−0.06	0.01	0.96	−0.05	0	1.3	0.04
rs1645732	FYB | C9	−0.21	0.56	1.1	0.0496	0.21	−4.5	−1.5	0.06
rs10059242	HTR4 | ADRB2	−0.90	1.8	1.5	0.04	0.89	−2.1	−2.0	0.04
rs12111032	HLA-C | HLA-B	−0.04	−0.17	**0.78**	**<0.0001**	0.04	0.23	−0.27	0.45
rs9275383	HLA-DQB1 | HLA-DQA2	−0.28	0.78	2.3	0.002	NA	4	NA	0.99
rs2551698	GSR	3.4	0.07	−0.60	0.003	−4.5	0.41	0.32	0.43
rs12543466	ANGPT1 | RSPO2	−0.17	0.84	0.53	0.26	0.18	−1	−1.6	0.03
rs597587	MYEOV | CCND1	2.9	−0.25	**−1.1**	**<0.0001**	−2.9	1.0	2.0	0.008
rs1586910	DCN | BTG1	0.12	−0.75	−0.14	0.38	−0.13	0.64	1.1	0.006
rs6018027	SRC	2.7	−1.8	−0.26	0.08	−3.4	0.35	0.65	0.04
rs4969754	RPS6KA3 | CNKSR2	6.6	0.29	−0.53	0.02	−4.9	−0.09	0.03	0.94

The estimates and p-values are obtained using the usual logistic regression model with the clinically diagnosed status as an outcome variable (model 3). Magnitude of bias is estimated using approximations (A11.1)–(A15.1) assuming the true model is (1) and using approximations (A19.1)–(A25.1) assuming the true model is (2). Boldfaced values are the results with a p-value < 0.05/13.

For three SNPs, rs401904, rs1748383, and rs12692222, the estimates in the usual logistic model (3) are nearly zero, while biases estimated assuming model (1) is true are nearly zero as well. This is correct with the theoretical observations that the null effect estimate based on the logistic regression can be unbiased.

## Discussion

We derived a simple and general approximation to bias in G×E parameter estimates when multiple pathologic mechanisms present with the same set of symptoms. The approximation to the bias relies on the estimates of the frequencies of the pathologic disease state within the clinical diagnosis. The approximation that we derived complements a recent study (Lobach et al., 2018) where we developed a pseudolikelihood method to incorporate uncertainty about the clinical–pathological diagnosis relationship, where the solution requires optimization of a complex nonlinear function. The approximation that we derived provides a simple formula that is intuitive and easy to apply.

We observed that parameter estimates could be substantially biased when the presence of the nuisance pathology is ignored. This observation has been also made by Manchia et al. (2013), where the simulation studies and data analyses on the main effect of genotype showed that the risk parameter estimates attributable to the genetics could be largely underestimated.

We define the bias to be a difference between the estimates obtained with the clinically diagnosed disease status as an outcome variable and the estimates with respect to the disease states. Within the notation of this study, bias is the difference between Γ and *B*. For example, from approximations (4)–(7), we see that the bias of a coefficient is a function of the true value of the coefficient, frequencies of the disease states within the clinical diagnosis, and also values of the other coefficients. Often, but not always, the null effect is correctly estimated, as we describe in the Remarks. When the proportions of the disease state of interest within the clinical diagnosis are the same in subpopulations by the environmental variable, in large samples, the estimates with the clinical diagnosis as an outcome variable are nearly unbiased. Even then, in practical settings, however, the estimates that ignore the presence of the nuisance disease might still have notable bias.

In our analysis of AD, the reliability study is based on 1,121 carriers of the ApoE ε4 allele and 1,331 noncarriers of the ApoE ε4 allele. Hence, we suppose that the clinical–pathological diagnosis relationship is estimated reasonably reliably well. Moreover, the reliability study is performed on the same patient population, i.e. patients followed up by the AD centers. There is a substantial, although not exactly known, overlap between the set with genotypes and the reliability set. In general, we advocate sensitivity analyses that examine potential differences in the parameter estimates due to misspecifications of frequencies of the pathologic diagnosis within the set of clinically defined cases.

Our derivations are based on a logistic model with a linear disease risk function. The same line of arguments might be extended to more complex relationships when, e.g., the link is not logit and when the interaction is nonlinear (Wu and Cui, 2013; Wu et al., 2018). These extensions might be the natural extensions of the current study. Another possible extension is to consider the setting when the disease states are the stages of the disease. Then the disease stage can be an outcome variable in a multilevel proportional logistic regression model.

In the context of gene–environment interaction analyses, model selection is often needed. While the current study does not provide a mechanism for model selection, in our previous work, we devised a pseudolikelihood model to correct for bias (Lobach et al., 2018) that can be used as a basis for model selection.

Though in the data analyses, we extracted the 113 SNPs for a better illustration of the application of proposed methods, the current method can be easily extended to all SNPs measured in a GWAS. Many of the SNPs would probably be not associated with the disease status, however, since as we see in the data analyses and theoretical derivations, null effects are often correctly estimated.

While our study is motivated by a specific example of AD, the application of the approximation that we derived is readily applicable to other diseases. For example, recent studies report that the underlying biologic mechanisms of breast cancer vary by expression of progesterone and estrogen measured in the ER/PR/HER2 status. Frequencies of subtypes can be estimated based on the SEER database (https://seer.cancer.gov/). Another possible application is when disease states of cancer are estimated by the mutation patterns available in the Cancer Genome Atlas.

## Data Availability

The data analyzed in this work are available at the Database of Genotypes and Phenotypes, study accession number phs000372.v1.p1 (https://www.ncbi.nlm.nih.gov/projects/gap/cgi-bin/study.cgi?study_id=phs000372.v1.p1), and phenotypic collection is at the National Alzheimer’s Coordinating Center (https://www.alz.washington.edu/).

## Ethics Statement

The study is based on dataset downloaded from dbGaP database. The data are completely de-identified and identifiers are not available to the study researchers.

## Author Contributions

IL conceived the study, wrote the manuscript, derived the approximation, performed simulation studies and data analyses.IK supported the derivations of the approximations, simulation studies and data analyses; and reviewed the manuscript. AA mapped the genetic variants into subsets, supervised the analyses and interpretation of the results. SL contributed to the derivations, performed simulation studies and reviewed the manuscript. LZ provided overall supervision of the study.

## Funding

IL is supported by grant # 5R21AG043710-02 from the National Institutes of Health.

## Conflict of Interest Statement

The authors declare that the research was conducted in the absence of any commercial or financial relationships that could be construed as a potential conflict of interest.
