# Supplementary material for: A Simple Approximation to Bias in Gene–Environment Interaction Estimates When a Case Might Not Be the Case
Source: Front Genet. 2019 Oct 9;10:886. doi: 10.3389/fgene.2019.00886 (PMC6812609; doi:10.3389/fgene.2019.00886)
Supplement: Supplementary file 1 [file Table_1.docx]

Online methods for

“**A simple approximation to bias in gene-environment interaction estimates when a case might not be the case**”

Iryna Lobach, Inyoung Kim, Alexander Alekseyenko, Siarhei Lobach, Li Zhang

July 11, 2019

**APPENDIX**

**DERIVATION OF THE APPROXIMATION**

The observed data are collected using retrospective case-control sampling design, i.e. the genetic and environmental variables are sampled conditionally on the clinical diagnosis. The likelihood function of the observed data is based on the probability $[G,X|D^{CL}]$ and hence we define $Q_{B,B^{*}}^{TRUE}\left( D^{CL},G,X \right)=\frac{\sum_{d^{*}} \mathrm{pr}[D^{CL}|D=d^{*},X]\times\mathrm{pr}_{B,B^{*}}\left[ D=d^{*} | G,X \right]\times\mathrm{pr}[G,X]}{\sum_{d^{*},g^{*},x^{*},z^{*}} \mathrm{pr}[D^{CL}|D=d^{*},X=x^{*}]\times\mathrm{pr}_{B,B^{*}}\left[ D=d^{*} | G=g^{*},X=x^{*} \right]\times\mathrm{pr}[G=g^{*},X=x^{*}]}$. (4)

The usual logistic regression model with the clinically diagnosed disease status as the outcome variable is

$Q_{\Gamma}^{FALSE}(D^{CL},G,X)=\mathrm{pr}_{\Gamma}\left[ D^{CL} | G,X \right]$, (5)

We are interested to find an analytic solution that relates parameters $\Gamma$from the misspecified model (5) to the parameters $B$and $B^{*}$ from the *true* model (4).

The next steps are motivated by the developments in Kullback (1959), Neuhaus (1999). Kullback (1959) showed that parameters $\Gamma$estimated in the misspesified model (4) converge to values that minimize the Kullback-Leibler divergence between the *true* and false models with expectations taken with respect to the *true* model, i.e.

$\Gamma=argmin\left( E_{X,G}\left[ E_{D^{CL}|X,G}\log\left\{ \frac{Q_{B}(D^{CL},G,X)}{Q_{B,B^{*}}\left( D^{CL},G,X \right)} \right\} \right] \right)$. (5)

We first examine the true model (1) and misspecified model (3).

We first derive an approximation to the relationship between the parameters of the misspecified model (3) to the parameters of the true model (1).

Derivative of the Kullback-Leibler divergence (5) with respect to parameters of the misspecified model are

$${-E}_{X,G,Z}\left[ \frac{\mathrm{pr}_{B}\left( D^{CL}=0 | G,X,Z \right)}{Q_{\Gamma}(D^{CL}=0,G,X,Z)}\times\frac{\partial}{\partial\Gamma}Q_{\Gamma}\left( D^{CL}=0 | G,X,Z \right)+\frac{\mathrm{pr}_{B}\left( D^{CL}=1 | G,X,Z \right)}{Q_{\Gamma}(D^{CL}=1,G,X,Z)}\times\frac{\partial}{\partial\Gamma}Q_{\Gamma}\left( D^{CL}=1 | G,X,Z \right) \right].$$

Define $K(g,x,z;\Gamma)=\frac{\exp\left\{ \gamma_{0}+\gamma_{G}\times g+\gamma_{X}\times x+\gamma_{Z}\times z+\gamma_{G\times X}\times g\times x \right\}}{(1+\exp\left\{ \gamma_{0}+\gamma_{G}\times g+\gamma_{X}\times x+\gamma_{Z}\times z+\gamma_{G\times X}\times g\times x \right\})^{2}}$, then taking derivatives of Kullback-Leibler divergence (5) with respect to $\gamma_{0},\gamma_{G},\gamma_{X},\gamma_{Z},\gamma_{G\times X}$ we arrive to the following system of equations

$E_{G,X,Z}\left[ K(g,x,z;\Gamma)\times\left\{ \frac{\mathrm{pr}_{B}\left( D^{CL}=0 | G,X,Z \right)}{\mathrm{pr}_{\Gamma}(D^{CL}=0|G,X,Z)}-\frac{\mathrm{pr}_{B}\left( D^{CL}=1 | G,X,Z \right)}{\mathrm{pr}_{\Gamma}(D^{CL}=1|G,X,Z)} \right\} \right]=0$; (A1)

$E_{G,X,Z}\left[ X\times K(g,x,z;\Gamma)\times\left\{ \frac{\mathrm{pr}_{B}\left( D^{CL}=0 | G,X,Z \right)}{\mathrm{pr}_{\Gamma}(D^{CL}=0|G,X,Z)}-\frac{\mathrm{pr}_{B}\left( D^{CL}=1 | G,X,Z \right)}{\mathrm{pr}_{\Gamma}(D^{CL}=1|G,X,Z)} \right\} \right]=0$;

$$E_{G,X,Z}\left[ Z\times K(g,x,z;\Gamma)\times\left\{ \frac{\mathrm{pr}_{B}\left( D^{CL}=0 | G,X,Z \right)}{\mathrm{pr}_{\Gamma}(D^{CL}=0|G,X,Z)}-\frac{\mathrm{pr}_{B}\left( D^{CL}=1 | G,X,Z \right)}{\mathrm{pr}_{\Gamma}(D^{CL}=1|G,X,Z)} \right\} \right]=0;$$

$E_{G,X,Z}\left[ G\times K(g,x,z;\Gamma)\times\left\{ \frac{\mathrm{pr}_{B}\left( D^{CL}=0 | G,X,Z \right)}{\mathrm{pr}_{\Gamma}(D^{CL}=0|G,X,Z)}-\frac{\mathrm{pr}_{B}\left( D^{CL}=1 | G,X,Z \right)}{\mathrm{pr}_{\Gamma}(D^{CL}=1|G,X,Z)} \right\} \right]=0$;

$$E_{G,X,Z}\left[ G\times X\times K(g,x,z;\Gamma)\times\left\{ \frac{\mathrm{pr}_{B}\left( D^{CL}=0 | G,X,Z \right)}{\mathrm{pr}_{\Gamma}(D^{CL}=0|G,X,Z)}-\frac{\mathrm{pr}_{B}\left( D^{CL}=1 | G,X,Z \right)}{\mathrm{pr}_{\Gamma}(D^{CL}=1|G,X,Z)} \right\} \right]=0.$$

Values of $\Gamma$ such that

$\frac{\mathrm{pr}_{B}\left( D^{CL}=0 | G,X,Z \right)}{\mathrm{pr}_{\Gamma}(D^{CL}=0|G,X,Z)}=\frac{\mathrm{pr}_{B}\left( D^{CL}=1 | G,X,Z \right)}{\mathrm{pr}_{\Gamma}\left( D^{CL}=1 | G,X,Z \right)}=1$ (A2)

for all $G,X,Z$ solve the system of equations (A2). Hence values of $\Gamma$ for which$\mathrm{pr}_{B}\left( D^{CL}=0 | G,X,Z \right)=\mathrm{pr}_{\Gamma}(D^{CL}=0|G,X,Z)$ and $\mathrm{pr}_{B}\left( D^{CL}=1 | G,X,Z \right)=\mathrm{pr}_{\Gamma}(D^{CL}=1|G,X,Z)$ for any$G,X,Z$ solve the system of equations (A2).

We first consider a setting with $\beta_{Z}=0$. By definition, $\gamma_{0}={logit\{pr}_{\Gamma}\left( D^{CL}=1 | G=0,X=0 \right)\}$. Because values of $\Gamma$ for which $\mathrm{pr}_{B}\left( D^{CL}=1 | G,X \right)=\mathrm{pr}_{\Gamma}(D^{CL}=1|G,X)$ solve the system of equations (7),

$\gamma_{0}=\mathrm{logit}\left\{ \mathrm{pr}_{B}\left( D^{CL}=1 | G=0,X=0 \right) \right\}$ =$\log\left\{ \frac{\mathcal{S}\left( 0 \right)+e^{\beta_{0}}}{1-\mathcal{S}\left( 0 \right)} \right\}=$ $H_{0}\left\{ \beta_{0}\mathcal{,S}(0) \right\}$. (A3)

In the next equations we apply the same arguments for the other parameters.

$\gamma_{X}={logit\{pr}_{\Gamma}\left( D^{CL}=1 | G=0,X=1 \right)\}-{logit\{pr}_{\Gamma}\left( D^{CL}=1 | G=0,X=0 \right)\}={logit\{pr}_{B}\left( D^{CL}=1 | G=0,X=1 \right)\}-{logit\{pr}_{B}\left( D^{CL}=1 | G=0,X=0 \right)\}= \log\left\{ \frac{\mathcal{S}\left( 1 \right)+e^{\beta_{0}+\beta_{X}}}{1-\mathcal{S}\left( 1 \right)} \right\}-\log\left\{ \frac{\mathcal{S}\left( 0 \right)+e^{\beta_{0}}}{1-\mathcal{S}\left( 0 \right)} \right\}=H_{X}\left\{ \beta_{0},\beta_{X}\mathcal{,S}\left( 0 \right)\mathcal{,S}(1) \right\}$; (A4)

$\gamma_{G}={logit\{pr}_{\Gamma}\left( D^{CL}=1 | G=1,X=0 \right)\}-{logit\{pr}_{\Gamma}\left( D^{CL}=1 | G=0,X=0 \right)\}={logit\{pr}_{B}\left( D^{CL}=1 | G=1,X=0 \right)\}-{logit\{pr}_{B}\left( D^{CL}=1 | G=0,X=0 \right)\}=\log\left\{ \frac{\mathcal{S}\left( 0 \right)+e^{\beta_{0}+\beta_{G}}}{1-\mathcal{S}\left( 0 \right)} \right\}-\log\left\{ \frac{\mathcal{S}\left( 0 \right)+e^{\beta_{0}}}{1-\mathcal{S}\left( 0 \right)} \right\}= H_{G}\left\{ \beta_{0},\beta_{G}\mathcal{,S}(0) \right\}$; (A5)

$\gamma_{G\times X}={logit\{pr}_{\Gamma}\left( D^{CL}=1 | G=1,X=1 \right)\}-{logit\{pr}_{\Gamma}\left( D^{CL}=1 | G=1,X=0 \right)\}-{logit\{pr}_{\Gamma}\left( D^{CL}=1 | G=0,X=1 \right)\}+{logit\{pr}_{\Gamma}\left( D^{CL}=1 | G=0,X=0 \right)\}={logit\{pr}_{B}\left( D^{CL}=1 | G=1,X=1 \right)\}-{logit\{pr}_{B}\left( D^{CL}=1 | G=1,X=0 \right)\}-{logit\{pr}_{B}\left( D^{CL}=1 | G=0,X=1 \right)\}+{logit\{pr}_{B}\left( D^{CL}=1 | G=0,X=0 \right)\}=\log\left\{ \frac{\mathcal{S}\left( 1 \right)+e^{\beta_{0}+\beta_{X}+\beta_{G}+\beta_{G\times X}}}{1-\mathcal{S}\left( 1 \right)} \right\}-\log\left\{ \frac{\mathcal{S}\left( 0 \right)+e^{\beta_{0}+\beta_{G}}}{1-\mathcal{S}\left( 0 \right)} \right\}-\log\left\{ \frac{\mathcal{S}\left( 1 \right)+e^{\beta_{0}+\beta_{X}}}{1-\mathcal{S}\left( 1 \right)} \right\}+\log\left\{ \frac{\mathcal{S}\left( 0 \right)+e^{\beta_{0}}}{1-\mathcal{S}\left( 0 \right)} \right\}=H_{G\times X}\left\{ \beta_{0},\beta_{X},\beta_{G},\beta_{G\times X}\mathcal{,S}\left( 0 \right)\mathcal{,S}(1) \right\}$. (A6)

While the equations (A2)-(A6) describe the relationship between the parameters $\Gamma$ and $B$, it is not clear what the relationship is. Taylor series expansion around 0 provides convenient approximation in the form

$\gamma_{0}\approx H_{0}\left\{ \beta_{0}=0,\mathcal{S}(0) \right\}+\beta_{0}\times\left. \frac{\partial H_{0}\left\{ \beta_{0}\mathcal{,S}(0) \right\}}{\partial\beta_{0}} \right|_{\beta_{0}=0}\approx\log\left\{ \frac{1+\mathcal{S}\left( 0 \right)}{1-\mathcal{S}\left( 0 \right)} \right\}+\frac{1}{1+\mathcal{S}\left( 0 \right)}\times\beta_{0};$ (A7)

$\gamma_{X}\approx H_{X}\left\{ \beta_{0},\beta_{X}=0,\mathcal{S}(0),\mathcal{S}(1) \right\}+\beta_{X}\times\left. \frac{\partial H_{X}\left\{ \beta_{0},\beta_{X}\mathcal{,S}(0),\mathcal{S}(1) \right\}}{\partial\beta_{X}} \right|_{\beta_{X}=0}=\log\left\{ \frac{\mathcal{S}\left( 1 \right)+e^{\beta_{0}}}{1-\mathcal{S}\left( 1 \right)} \right\}-\log\left\{ \frac{\mathcal{S}\left( 0 \right)+e^{\beta_{0}}}{1-\mathcal{S}\left( 0 \right)} \right\}+\frac{e^{\beta_{0}}}{e^{\beta_{0}}\mathcal{+S}\left( 1 \right)}\times\beta_{X}$; (A8)

$\gamma_{G}\approx H_{G}\left\{ \beta_{0},\beta_{G}=0,\mathcal{S}(0) \right\}+\beta_{G}\times\left. \frac{\partial H_{G}\left\{ \beta_{0},\beta_{G}\mathcal{,S}(0) \right\}}{\partial\beta_{G}} \right|_{\beta_{G}=0}=\frac{e^{\beta_{0}}}{e^{\beta_{0}}\mathcal{+S}\left( 0 \right)}\times\beta_{G};$ (A9)

$\gamma_{G\times X}\approx H_{G\times X}\left\{ \beta_{0},\beta_{X},\beta_{G},\beta_{G\times X}=0,\mathcal{S}(0),\mathcal{S}(1) \right\}+\beta_{G\times X}\times\left. \frac{\partial H_{G\times X}\left\{ \beta_{0},\beta_{X},\beta_{G},\beta_{G\times X}\mathcal{,S}(0),\mathcal{S}(1) \right\}}{\partial\beta_{G\times X}} \right|_{\beta_{G\times X}=0}=\log\left[ \frac{\mathcal{S}(1)\times e^{\beta_{0}+\beta_{X}+\beta_{G}}}{1+\mathcal{S}(1)\times e^{\beta_{0}+\beta_{X}+\beta_{G}}} \right]-\log\left[ \frac{\mathcal{S}(0)\times e^{\beta_{0}+\beta_{G}}}{1+\mathcal{S}(0)\times e^{\beta_{0}+\beta_{G}}} \right]-\log\left[ \frac{\mathcal{S}(1)\times e^{\beta_{0}+\beta_{X}}}{1+\mathcal{S}(1)\times e^{\beta_{0}+\beta_{X}}} \right]+\log\left[ \frac{\mathcal{S}(0)\times e^{\beta_{0}}}{1+\mathcal{S}(0)\times e^{\beta_{0}}} \right]+\frac{1}{1+\mathcal{S}(1)\times e^{\beta_{0}+\beta_{X}+\beta_{G}}}\times\beta_{G\times X}$. (A10)

It can be easily seen that when a variable $Z$ is included in the model,

$\gamma_{0}\approx\log\left\{ \frac{\mathcal{S}\left( 0 \right)+e^{\beta_{0}}}{1-\mathcal{S}\left( 0 \right)} \right\}$; (A11)

$\gamma_{X}\approx0.5\times\sum_{z^{*}} \left[ \log\left\{ \frac{\mathcal{S}\left( 1 \right)+e^{\beta_{0}+\beta_{Z}\times z^{*}+\beta_{X}}}{1-\mathcal{S}\left( 1 \right)} \right\}-\log\left\{ \frac{\mathcal{S}\left( 0 \right)+e^{\beta_{0}+\beta_{Z}\times z^{*}}}{1-\mathcal{S}\left( 0 \right)} \right\} \right];$ (A12)

$\gamma_{G}\approx0.5\times\sum_{z^{*}} \left[ \log\left\{ \frac{\mathcal{S}\left( 0 \right)+e^{\beta_{0}+\beta_{Z}\times z^{*}+\beta_{G}}}{1-\mathcal{S}\left( 0 \right)} \right\}-\log\left\{ \frac{\mathcal{S}\left( 0 \right)+e^{\beta_{0}+\beta_{Z}\times z^{*}}}{1-\mathcal{S}\left( 0 \right)} \right\} \right]$; (A13)

$\gamma_{Z}\approx0.25\times\sum_{g^{*},x^{*}} \left[ \log\left\{ \frac{\mathcal{S}\left( x^{*} \right)+e^{\beta_{0}+\beta_{Z}+\beta_{G}\times g^{*}+\beta_{X}\times x^{*}+\beta_{G\times X}\times g^{*}\times x^{*}}}{1-\mathcal{S}\left( x^{*} \right)} \right\}-\log\left\{ \frac{\mathcal{S}\left( x^{*} \right)+e^{\beta_{0}+\beta_{G}\times g^{*}+\beta_{X}\times x^{*}+\beta_{G\times X}\times g^{*}\times x^{*}}}{1-\mathcal{S}\left( x^{*} \right)} \right\} \right];$ (A14)

$\gamma_{G\times X}\approx0.5\times\sum_{z^{*}} \left[ \log\left\{ \frac{\mathcal{S}\left( 1 \right)+e^{\beta_{0}+\beta_{Z}\times z^{*}+\beta_{X}+\beta_{G}+\beta_{G\times X}}}{1-\mathcal{S}\left( 1 \right)} \right\}-\log\left\{ \frac{\mathcal{S}\left( 0 \right)+e^{\beta_{0}+\beta_{Z}\times z^{*}+\beta_{G}}}{1-\mathcal{S}\left( 0 \right)} \right\}-\log\left\{ \frac{\mathcal{S}\left( 1 \right)+e^{\beta_{0}+\beta_{Z}\times z^{*}+\beta_{X}}}{1-\mathcal{S}\left( 1 \right)} \right\}+\log\left\{ \frac{\mathcal{S}\left( 0 \right)+e^{\beta_{0}+\beta_{Z}\times z^{*}}}{1-\mathcal{S}\left( 0 \right)} \right\} \right]$ . (A15)

With Taylor series expansion the system of equations becomes (A11)-(A15)

$\gamma_{0}\approx\log\left\{ \frac{1+\mathcal{S}\left( 0 \right)}{1-\mathcal{S}\left( 0 \right)} \right\}+\frac{1}{1+\mathcal{S}\left( 0 \right)}\times\beta_{0}$; (A11.1)

$\gamma_{X}\approx0.5\times\sum_{z^{*}} \left[ \log\left\{ \frac{\mathcal{S}\left( 1 \right)+e^{\beta_{0}+\beta_{Z}\times z^{*}}}{1-\mathcal{S}\left( 1 \right)} \right\}-\log\left\{ \frac{\mathcal{S}\left( 0 \right)+e^{\beta_{0}+\beta_{Z}\times z^{*}}}{1-\mathcal{S}\left( 0 \right)} \right\}+\frac{e^{\beta_{0}+\beta_{Z}\times z^{*}}}{e^{\beta_{0}+\beta_{Z}\times z^{*}}\mathcal{+S}\left( 1 \right)}\times\beta_{X} \right]$; (A12.1)

$\gamma_{G}\approx0.5\times\sum_{z^{*}} \left[ \frac{e^{\beta_{0}+\beta_{Z}\times z^{*}}}{e^{\beta_{0}+\beta_{Z}\times z^{*}}\mathcal{+S}\left( 0 \right)}\times\beta_{G} \right]$; (A13.1)

$\gamma_{Z}\approx0.25\times\sum_{g^{*},x^{*}} \left[ \log\left\{ \frac{\mathcal{S}\left( x^{*} \right)+e^{\beta_{0}+\beta_{Z}+\beta_{G}\times g^{*}+\beta_{X}\times x^{*}+\beta_{G\times X}\times g^{*}\times x^{*}}}{1-\mathcal{S}\left( x^{*} \right)} \right\}-\log\left\{ \frac{\mathcal{S}\left( x^{*} \right)+e^{\beta_{0}+\beta_{G}\times g^{*}+\beta_{X}\times x^{*}+\beta_{G\times X}\times g^{*}\times x^{*}}}{1-\mathcal{S}\left( x^{*} \right)} \right\} \right]$; (A14.1)

$\gamma_{G\times X}\approx0.5\times\sum_{z^{*}} \left[ \log\left\{ \frac{\mathcal{S}\left( 1 \right)+e^{\beta_{0}+\beta_{Z}\times z^{*}+\beta_{X}+\beta_{G}+\beta_{G\times X}}}{1-\mathcal{S}\left( 1 \right)} \right\}-\log\left\{ \frac{\mathcal{S}\left( 0 \right)+e^{\beta_{0}+\beta_{Z}\times z^{*}+\beta_{G}}}{1-\mathcal{S}\left( 0 \right)} \right\}-\log\left\{ \frac{\mathcal{S}\left( 1 \right)+e^{\beta_{0}+\beta_{Z}\times z^{*}+\beta_{X}}}{1-\mathcal{S}\left( 1 \right)} \right\}+\log\left\{ \frac{\mathcal{S}\left( 0 \right)+e^{\beta_{0}+\beta_{Z}\times z^{*}}}{1-\mathcal{S}\left( 0 \right)} \right\} \right].$ (A15.1)

We next suppose that the true model is (2), while the parameters are estimated based on a misspecified model (3).

By definition, $\gamma_{0}=\mathrm{logit}\{\mathrm{pr}_{\Gamma}\left( D^{CL}=1|G=0,X=0 \right)\}$. Because values of parameters $\Gamma$ for which $\mathrm{pr}_{\Gamma}\left( D^{CL}=1|G=0,X=0 \right)=\mathrm{pr}_{B,B^{*}}\left( D^{CL}=1|G=0,X=0 \right)\}$ minimize the Kullback-Leibler divergence (5), $\gamma_{0}=\mathrm{logit}\{\mathrm{pr}_{B,B^{*}}\left( D^{CL}=1|G=0,X=0 \right)\}$. Algebraic derivations show that then $\gamma_{0}=\log\left( e^{\beta_{0}}+e^{\beta_{0}^{*}} \right)$ and Taylor series expansion around zero arrives at an approximation $\gamma_{0}\approx\beta_{0}^{*}+\frac{1}{1+e^{\beta_{0}^{*}}}\times\beta_{0}.$ Similar derivations apply to the other parameters:

$\gamma_{G}=\mathrm{logit}\left\{ \mathrm{pr}_{\Gamma}\left( D^{CL}=1|G=1,X=0 \right) \right\}-\mathrm{logit}\left\{ \mathrm{pr}_{\Gamma}\left( D^{CL}=1|G=0,X=0 \right) \right\}=log \left( e^{\beta_{0}+\beta_{G}}+e^{\beta_{0}^{*}+\beta_{G}^{*}} \right)-\log\left( e^{\beta_{0}}+e^{\beta_{0}^{*}} \right)\approx\frac{e^{\beta_{0}}}{e^{\beta_{0}}+e^{\beta_{0}^{*}+\beta_{G}^{*}}}\times\beta_{G};$ (A16)

$\gamma_{X}=\mathrm{logit}\left\{ \mathrm{pr}_{\Gamma}\left( D^{CL}=1|G=0,X=1 \right) \right\}-\mathrm{logit}\left\{ \mathrm{pr}_{\Gamma}\left( D^{CL}=1|G=0,X=0 \right) \right\}=\log\left( e^{\beta_{0}+\beta_{X}}+e^{\beta_{0}^{*}+\beta_{X}^{*}} \right)-\log\left( e^{\beta_{0}}+e^{\beta_{0}^{*}} \right)\approx\frac{e^{\beta_{0}}}{e^{\beta_{0}}+e^{\beta_{0}^{*}+\beta_{X}^{*}}}\times\beta_{X};$ (A17)

$\gamma_{G\times X}=\mathrm{logit}\left\{ \mathrm{pr}_{\Gamma}\left( D^{CL}=1|G=1,X=1 \right) \right\}-\mathrm{logit}\left\{ \mathrm{pr}_{\Gamma}\left( D^{CL}=1|G=0,X=1 \right) \right\}-\mathrm{logit}\left\{ \mathrm{pr}_{\Gamma}\left( D^{CL}=1|G=1,X=0 \right) \right\}+\mathrm{logit}\left\{ \mathrm{pr}_{\Gamma}\left( D^{CL}=1|G=0,X=0 \right) \right\}=\log\left( e^{\beta_{0}+\beta_{G}+\beta_{X}+\beta_{G\times X}}+e^{\beta_{0}^{*}+\beta_{G}^{*}+\beta_{X}^{*}+\beta_{G\times X}^{*}} \right)-\log\left( e^{\beta_{0}+\beta_{X}}+e^{\beta_{0}^{*}+\beta_{X}^{*}} \right)-\log\left( e^{\beta_{0}+\beta_{G}}+e^{\beta_{0}^{*}+\beta_{G}^{*}} \right)+\log\left( e^{\beta_{0}}+e^{\beta_{0}^{*}} \right)\approx\log\left( e^{\beta_{0}+\beta_{G}+\beta_{X}}+e^{\beta_{0}^{*}+\beta_{G}^{*}+\beta_{X}^{*}+\beta_{G\times X}^{*}} \right)-\log\left( e^{\beta_{0}+\beta_{X}}+e^{\beta_{0}^{*}+\beta_{X}^{*}} \right)-\log\left( e^{\beta_{0}+\beta_{G}}+e^{\beta_{0}^{*}+\beta_{G}^{*}} \right)+\log\left( e^{\beta_{0}}+e^{\beta_{0}^{*}} \right)+\frac{e^{\beta_{0}+\beta_{G}+\beta_{X}}}{e^{\beta_{0}+\beta_{G}+\beta_{X}}+e^{\beta_{0}^{*}+\beta_{G}^{*}+\beta_{X}^{*}+\beta_{G\times X}^{*}}}\times\beta_{G\times X}.$ (A18)

When an environmental variable $Z$ that does not interact with the genotype is in the model, then the formulas are as follows:

When an environmental variable $Z$ that does not interact with the genotype is in the model, then the formulas are as follows:

$\gamma_{0}=\log\left( e^{\beta_{0}}+e^{\beta_{0}^{*}} \right)$; (A19)

$\gamma_{G}=0.5\times\sum_{z^{*}} \left\{ \log\left( e^{\beta_{0}+\beta_{G}+\beta_{Z}\times z^{*}}+e^{\beta_{0}^{*}+\beta_{G}^{*}+\beta_{Z}\times z^{*}} \right)-\log\left( e^{\beta_{0}+\beta_{Z}\times z^{*}}+e^{\beta_{0}^{*}+\beta_{Z}\times z^{*}} \right) \right\};$ (A20)

$\gamma_{X}=0.5\times\sum_{z^{*}} \left\{ \log\left( e^{\beta_{0}+\beta_{X}+\beta_{Z}\times z^{*}}+e^{\beta_{0}^{*}+\beta_{X}^{*}+\beta_{Z}^{*}\times z^{*}} \right)-\log\left( e^{\beta_{0}+\beta_{Z}\times z^{*}}+e^{\beta_{0}^{*}+\beta_{Z}^{*}\times z^{*}} \right) \right\};$ (A21)

$\gamma_{Z}=0.5\times\sum_{g^{*},x^{*}} \left\{ \log\left( e^{\beta_{0}+\beta_{Z}+\beta_{G}\times g^{*}+\beta_{X}\times x^{*}+\beta_{X\times G}\times x^{*}\times g^{*}}+e^{\beta_{0}^{*}+\beta_{Z}^{*}+\beta_{G}^{*}\times g^{*}+\beta_{X}^{*}\times x^{*}+\beta_{X\times G}^{*}\times x^{*}\times g^{*}} \right)-\log\left( e^{\beta_{0}+\beta_{G}\times g^{*}+\beta_{X}\times x^{*}+\beta_{X\times G}\times x^{*}\times g^{*}}+e^{\beta_{0}^{*}+\beta_{G}^{*}\times g^{*}+\beta_{X}^{*}\times x^{*}+\beta_{X\times G}^{*}\times x^{*}\times g^{*}} \right) \right\};$ (A22)

$\gamma_{G\times X}=0.5\times\sum_{z^{*}} \left\{ \log\left( e^{\beta_{0}+\beta_{G}+\beta_{X}+\beta_{G\times X}+\beta_{Z}\times z^{*}}+e^{\beta_{0}^{*}+\beta_{G}^{*}+\beta_{X}^{*}+\beta_{G\times X}^{*}+\beta_{Z}\times z^{*}} \right)-\log\left( e^{\beta_{0}+\beta_{X}+\beta_{Z}\times z^{*}}+e^{\beta_{0}^{*}+\beta_{X}^{*}+\beta_{Z}\times z^{*}} \right)-\log\left( e^{\beta_{0}+\beta_{G}+\beta_{Z}\times z^{*}}+e^{\beta_{0}^{*}+\beta_{G}^{*}+\beta_{Z}\times z^{*}} \right)+\log\left( e^{\beta_{0}+\beta_{Z}\times z^{*}}+e^{\beta_{0}^{*}+\beta_{Z}\times z^{*}} \right) \right\} -\log\left( e^{\beta_{0}+\beta_{X}+\beta_{Z}\times z^{*}}+e^{\beta_{0}^{*}+\beta_{X}^{*}+\beta_{Z}\times z^{*}} \right)-\log\left( e^{\beta_{0}+\beta_{G}+\beta_{Z}\times z^{*}}+e^{\beta_{0}^{*}+\beta_{G}^{*}+\beta_{Z}\times z^{*}} \right)+\log\left( e^{\beta_{0}+\beta_{Z}\times z^{*}}+e^{\beta_{0}^{*}+\beta_{Z}\times z^{*}} \right)\}.$ (A23)

With Taylor series expansion the system of equations (A19)-(A23) becomes

$\gamma_{0}\approx\beta_{0}^{*}+\frac{1}{1+e^{\beta_{0}^{*}}}\times\beta_{0}$; (A19.1)

$\gamma_{G}\approx0.5\times\sum_{z^{*}} \left\{ \frac{e^{\beta_{0}+\beta_{Z}\times z^{*}}}{e^{\beta_{0}+\beta_{Z}\times z^{*}}+e^{\beta_{0}^{*}+\beta_{G}^{*}+\beta_{Z}\times z^{*}}}\times\beta_{G} \right\} ; ;$ (A20.1)

$$\gamma_{X}\approx0.5\times$$

$\sum_{z^{*}} \left\{ \frac{e^{\beta_{0}+\beta_{Z}\times z^{*}}}{e^{\beta_{0}+\beta_{Z}\times z^{*}}+e^{\beta_{0}^{*}+\beta_{X}^{*}+\beta_{Z}\times z^{*}}}\times\beta_{X} \right\};$ (A21.1)

$\gamma_{Z}\approx0.5\times\sum_{z^{*}} \left\{ \log\left( e^{\beta_{0}+\beta_{Z}+\beta_{G}\times g^{*}+\beta_{X}\times x^{*}+\beta_{X\times G}\times x^{*}\times g^{*}}+e^{\beta_{0}^{*}+\beta_{X}^{*}+\beta_{G}^{*}\times g^{*}+\beta_{X}^{*}\times x^{*}+\beta_{X\times G}^{*}\times x^{*}\times g^{*}} \right)-\log\left( e^{\beta_{0}+\beta_{X}+\beta_{Z}\times z^{*}}+e^{\beta_{0}^{*}+\beta_{X}^{*}+\beta_{Z}\times z^{*}} \right)+\frac{e^{\beta_{0}+\beta_{Z}\times z^{*}}}{e^{\beta_{0}+\beta_{Z}\times z^{*}}+e^{\beta_{0}^{*}+\beta_{X}^{*}+\beta_{Z}\times z^{*}}}\times\beta_{X} \right\};$ (A22.1)

$\gamma_{G\times X}=0.5\times\sum_{z^{*}} \left\{ \log\left( e^{\beta_{0}+\beta_{G}+\beta_{X}+\beta_{Z}\times z^{*}}+e^{\beta_{0}^{*}+\beta_{G}^{*}+\beta_{X}^{*}+\beta_{G\times X}^{*}+\beta_{Z}\times z^{*}} \right)-\log\left( e^{\beta_{0}+\beta_{X}+\beta_{Z}\times z^{*}}+e^{\beta_{0}^{*}+\beta_{X}^{*}}+\beta_{Z}\times z^{*} \right)-\log\left( e^{\beta_{0}+\beta_{G}+\beta_{Z}\times z^{*}}+e^{\beta_{0}^{*}+\beta_{G}^{*}}+\beta_{Z}\times z^{*} \right)+\log\left( e^{\beta_{0}+\beta_{Z}\times z^{*}}+e^{\beta_{0}^{*}+\beta_{Z}\times z^{*}} \right)+\frac{e^{\beta_{0}+\beta_{G}+\beta_{X}+\beta_{Z}\times z^{*}}}{e^{\beta_{0}+\beta_{G}+\beta_{X}}+e^{\beta_{0}^{*}+\beta_{G}^{*}+\beta_{X}^{*}+\beta_{G\times X}^{*}+\beta_{Z}\times z^{*}}}\times\beta_{G\times X} \right\}.$ (A23.1)

| True model (1) | | Misspecified Model (3) | | | |
| --- | --- | --- | --- | --- | --- |
| Parameter | True Value | Parameter | Empirical average  estimate | Empirical SD | Approximation (A11)-(A15) |
| $\beta_{G}$ | 0.41 | $\gamma_{G}$ | 0.28 | 0.10 | 0.26 |
| $\beta_{\varepsilon4}$ | 2.08 | $\gamma_{\varepsilon4}$ | 1.80 | 0.09 | 1.30 |
| $\beta_{G\times\varepsilon4}$ | 0 | $\gamma_{G\times\varepsilon4}$ | 0.13 | 0.36 | 0.11 |
| $\beta_{Z_{1}}$ | 0 | $\gamma_{Z_{1}}$ | -0.00 | 0.05 | 0 |
| $\beta_{Z_{2}}$ | -0.08 | $\gamma_{Z_{2}}$ | -0.06 | 0.05 | -0.06 |

**Supplementary Table 1.** Estimates of coefficients obtained empirically as an average across 500 simulated datasets with 3,000 cases and 3,000 controls. The genetic variable is Bernoulli with frequency 0.1. The environmental variables $\varepsilon4,Z_{1}, Z_{2}$ are Bernoulli with frequencies 14%, 50%, and 52%, respectively. The *true* diagnosis is simulated based on the model (1) with coefficients $\beta_{0}=-1, \beta_{G}=0.41, \beta_{Z_{1}}=0, \beta_{Z_{2}}=-0.08, \beta_{\varepsilon4}=2.08$ and $\beta_{G\times\varepsilon4}=0.$The clinical-pathological diagnoses relationship is defined as $pr(D=1^{*}|D^{CL}=1,\varepsilon4-)=0.36$ and $pr(D=1^{*}|D^{CL}=1,\varepsilon4+)=0.06$. Coefficients $\gamma$s are estimated based on misspecified model (3).

| True model (2) | | Misspecified Model (3) | | | |
| --- | --- | --- | --- | --- | --- |
| Parameter | True Value | Parameter | Empirical average | Empirical SD | Approximation (A16)-(A22) |
| $\beta_{G}$ | -0.69 | $\gamma_{G}$ | -0.38 | 0.10 | -0.39 |
| $\beta_{\varepsilon4}$ | 1.3 | $\gamma_{\varepsilon4}$ | 1.08 | 0.08 | 1.08 |
| $\beta_{G\times\varepsilon4}$ | 0.10 | $\gamma_{G\times\varepsilon4}$ | -0.10 | 0.25 | -0.05 |
| $\beta_{Z_{1}}$ | 0.10 | $\gamma_{Z_{1}}$ | 0.10 | 0.05 | 0.10 |
| $\beta_{Z_{2}}$ | -0.08 | $\gamma_{Z_{2}}$ | -0.09 | 0.05 | -0.08 |

**Supplementary Table 2.** Estimates of coefficients obtained empirically as an average across 500 simulated datasets with 3,000 cases and 3,000 controls. Risk of the disease of interest is defined in a set of parameters $\beta_{0}, \beta_{G}, \beta_{Z_{1}},\beta_{Z_{2}}, \beta_{G\times\varepsilon4}$; while the risk of the nuisance disease is parametrized by $\beta_{0}^{*}, \beta_{G}^{*}, \beta_{\varepsilon4}^{*}, \beta_{G\times\varepsilon4}^{*}.$ Frequency of ApoE $\varepsilon$4 allele in the population is 14%. Variables $Z_{1}$ and $Z_{2}$ are Bernoulli with frequencies 0.50 and 0.52, respectively. $\beta_{0}=-1, \beta_{0}^{*}=-1.7, \beta_{G}=-0.69, \beta_{G}^{*}=0, \beta_{Z_{1}}=0.10, {\beta_{Z_{2}}=-0.083,\beta}_{\varepsilon4}=1.3, \beta_{\varepsilon4}^{*}=0.5, \beta_{G\times\varepsilon4}=1.099, \beta_{G\times\varepsilon4}^{*}=0, \mathrm{pr}\left( G=1 \right)=0.10.$Frequencies of the disease of interest and the nuisance disease are $pr(D=1)=24.8\%, pr(D=1^{*})=12.5\%, pr\left( D=1 | \varepsilon4+ \right)=43\%, pr\left( D=1^{*} | \varepsilon4+ \right)=16.1\%, pr\left( D=1 | \varepsilon4- \right)=20\%, pr\left( D=1^{*} | \varepsilon4- \right)=11.6\%.$ Frequency of the nuisance disease within the clinical diagnosis varies by ApoE4 status $pr(D=1^{*}|D^{CL}=1,\varepsilon4-$)=0.36 and $pr(D=1^{*}|D^{CL}=1,\varepsilon4+$)=0.06.

| True model (2) | | Misspecified Model (3) | | | |
| --- | --- | --- | --- | --- | --- |
| Parameter | True Value | Parameter | Empirical average | Empirical SD | Approximation (A16)-(A22) |
| $\beta_{G}$ | -0.69 | $\gamma_{G}$ | -0.33 | 0.14 | -0.39 |
| $\beta_{\varepsilon4}$ | 1.3 | $\gamma_{\varepsilon4}$ | 1.12 | 0.12 | 1.08 |
| $\beta_{G\times\varepsilon4}$ | 0.10 | $\gamma_{G\times\varepsilon4}$ | -0.10 | 0.30 | -0.05 |
| $\beta_{Z_{1}}$ | 0.10 | $\gamma_{Z_{1}}$ | 0.10 | 0.11 | 0.10 |
| $\beta_{Z_{2}}$ | -0.08 | $\gamma_{Z_{2}}$ | -0.03 | 0.12 | -0.08 |

**Supplementary Table 3.** Estimates of coefficients obtained empirically as an average across 500 simulated datasets with 2,000 cases and 1,000 controls. Risk of the disease of interest is defined in a set of parameters $\beta_{0}, \beta_{G}, \beta_{Z_{1}},\beta_{Z_{2}}, \beta_{G\times\varepsilon4}$; while the risk of the nuisance disease is parametrized by $\beta_{0}^{*}, \beta_{G}^{*}, \beta_{\varepsilon4}^{*}, \beta_{G\times\varepsilon4}^{*}.$ Frequency of ApoE $\varepsilon$4 allele in the population is 14%. Variables $Z_{1}$ and $Z_{2}$ are Bernoulli with frequencies 0.50 and 0.52, respectively. $\beta_{0}=-1, \beta_{0}^{*}=-1.7, \beta_{G}=-0.69, \beta_{G}^{*}=0, \beta_{Z_{1}}=0.10, {\beta_{Z_{2}}=-0.083,\beta}_{\varepsilon4}=1.3, \beta_{\varepsilon4}^{*}=0.5, \beta_{G\times\varepsilon4}=1.099, \beta_{G\times\varepsilon4}^{*}=0, \mathrm{pr}\left( G=1 \right)=0.10.$Frequencies of the disease of interest and the nuisance disease are $pr(D=1)=24.8\%, pr(D=1^{*})=12.5\%, pr\left( D=1 | \varepsilon4+ \right)=43\%, pr\left( D=1^{*} | \varepsilon4+ \right)=16.1\%, pr\left( D=1 | \varepsilon4- \right)=20\%, pr\left( D=1^{*} | \varepsilon4- \right)=11.6\%.$ Frequency of the nuisance disease within the clinical diagnosis varies by ApoE4 status $pr(D=1^{*}|D^{CL}=1,\varepsilon4-$)=0.36 and $pr(D=1^{*}|D^{CL}=1,\varepsilon4+$)=0.06.

| True model (2) | | Misspecified Model (3) | | | | | | | |
| --- | --- | --- | --- | --- | --- | --- | --- | --- | --- |
|  |  | Parameter | Approximation (A16)-(A22) | Rates of nuisance disease state are overestimated for both $\varepsilon4$ carriers and non-carriers | | Rates of nuisance disease state are underestimated for both $\varepsilon4$ carriers and non-carriers | | Rates of nuisance disease state are overestimated for $\varepsilon4$ carriers and underestimated fornon-carriers | |
| Parameter | True Value |  |  | Empirical average | Empirical SD | Empirical average | Empirical SD | Empirical average | Empirical SD |
| $\beta_{G}$ | -0.69 | $\gamma_{G}$ | -0.39 | -0.34 | 0.17 | -0.37 | 0.16 | -0.39 | 0.20 |
| $\beta_{\varepsilon4}$ | 1.3 | $\gamma_{\varepsilon4}$ | 1.08 | 1.14 | 0.14 | 1.12 | 0.15 | 1.13 | 0.19 |
| $\beta_{G\times\varepsilon4}$ | 0.10 | $\gamma_{G\times\varepsilon4}$ | -0.05 | -0.08 | 0.33 | -0.11 | 0.33 | -0.11 | 0.39 |
| $\beta_{Z_{1}}$ | 0.10 | $\gamma_{Z_{1}}$ | 0.10 | 0.10 | 0.07 | 0.11 | 0.09 | 0.10 | 0.10 |
| $\beta_{Z_{2}}$ | -0.08 | $\gamma_{Z_{2}}$ | -0.08 | -0.05 | 0.10 | -0.09 | 0.12 | -0.12 | 0.12 |

**Supplementary Table 3.** Estimates of coefficients obtained empirically as an average across 500 simulated datasets with 2,000 cases and 1,000 controls. Risk of the disease of interest is defined in a set of parameters $\beta_{0}, \beta_{G}, \beta_{Z_{1}},\beta_{Z_{2}}, \beta_{G\times\varepsilon4}$; while the risk of the nuisance disease is parametrized by $\beta_{0}^{*}, \beta_{G}^{*}, \beta_{\varepsilon4}^{*}, \beta_{G\times\varepsilon4}^{*}.$ Frequency of ApoE $\varepsilon$4 allele in the population is 14%. Variables $Z_{1}$ and $Z_{2}$ are Bernoulli with frequencies 0.50 and 0.52, respectively. $\beta_{0}=-1, \beta_{0}^{*}=-1.7, \beta_{G}=-0.69, \beta_{G}^{*}=0, \beta_{Z_{1}}=0.10, {\beta_{Z_{2}}=-0.083,\beta}_{\varepsilon4}=1.3, \beta_{\varepsilon4}^{*}=0.5, \beta_{G\times\varepsilon4}=1.099, \beta_{G\times\varepsilon4}^{*}=0, \mathrm{pr}\left( G=1 \right)=0.10.$Frequencies of the disease of interest and the nuisance disease are $pr(D=1)=24.8\%, pr(D=1^{*})=12.5\%, pr\left( D=1 | \varepsilon4+ \right)=43\%, pr\left( D=1^{*} | \varepsilon4+ \right)=16.1\%, pr\left( D=1 | \varepsilon4- \right)=20\%, pr\left( D=1^{*} | \varepsilon4- \right)=11.6\%.$ Frequency of the nuisance disease within the clinical diagnosis varies by ApoE4 status $pr(D=1^{*}|D^{CL}=1,\varepsilon4-$)=0.36 and $pr(D=1^{*}|D^{CL}=1,\varepsilon4+$)=0.06.


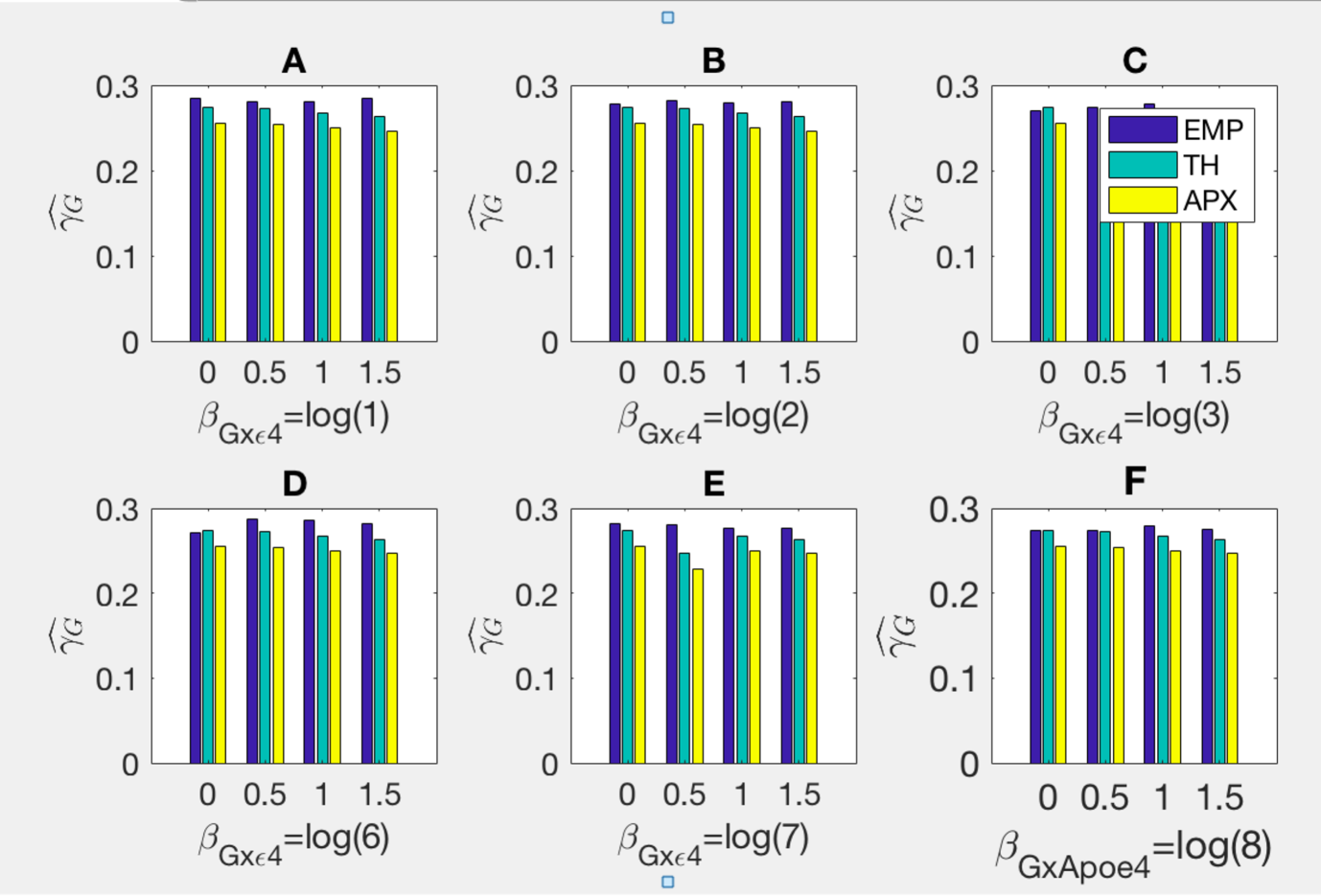


**Supplementary Figure 1A**: Estimates of $\gamma_{G}$ obtained empirically as an average across 500 simulated datasets (EMP) with 3,000 cases and 3,000 controls; using the theoretical formulae (A11)-(A15) (TH); and using the approximation (A11.1)-(A15.1) (APX). Coefficients $\gamma$s are estimated based on misspecified model (3).

The genetic variable is Bernoulli with frequency 0.1. The environmental variables $\varepsilon4,Z_{1}, Z_{2}$ are Bernoulli with frequencies 14%, 50%, and 52%, respectively. The *true* diagnosis is simulated based on the model (1) with coefficients $\beta_{0}=-1, \beta_{G}=0.41, \beta_{Z_{1}}=0, \beta_{Z_{2}}=-0.08, \beta_{\varepsilon4}=2.1$ and $\beta_{G\times\varepsilon4}=0.$The clinical-pathological diagnoses relationship is defined as $pr(D=1^{*}|D^{CL}=1,\varepsilon4-)=0.36$ and $pr(D=1^{*}|D^{CL}=1,\varepsilon4+)=0.06$.


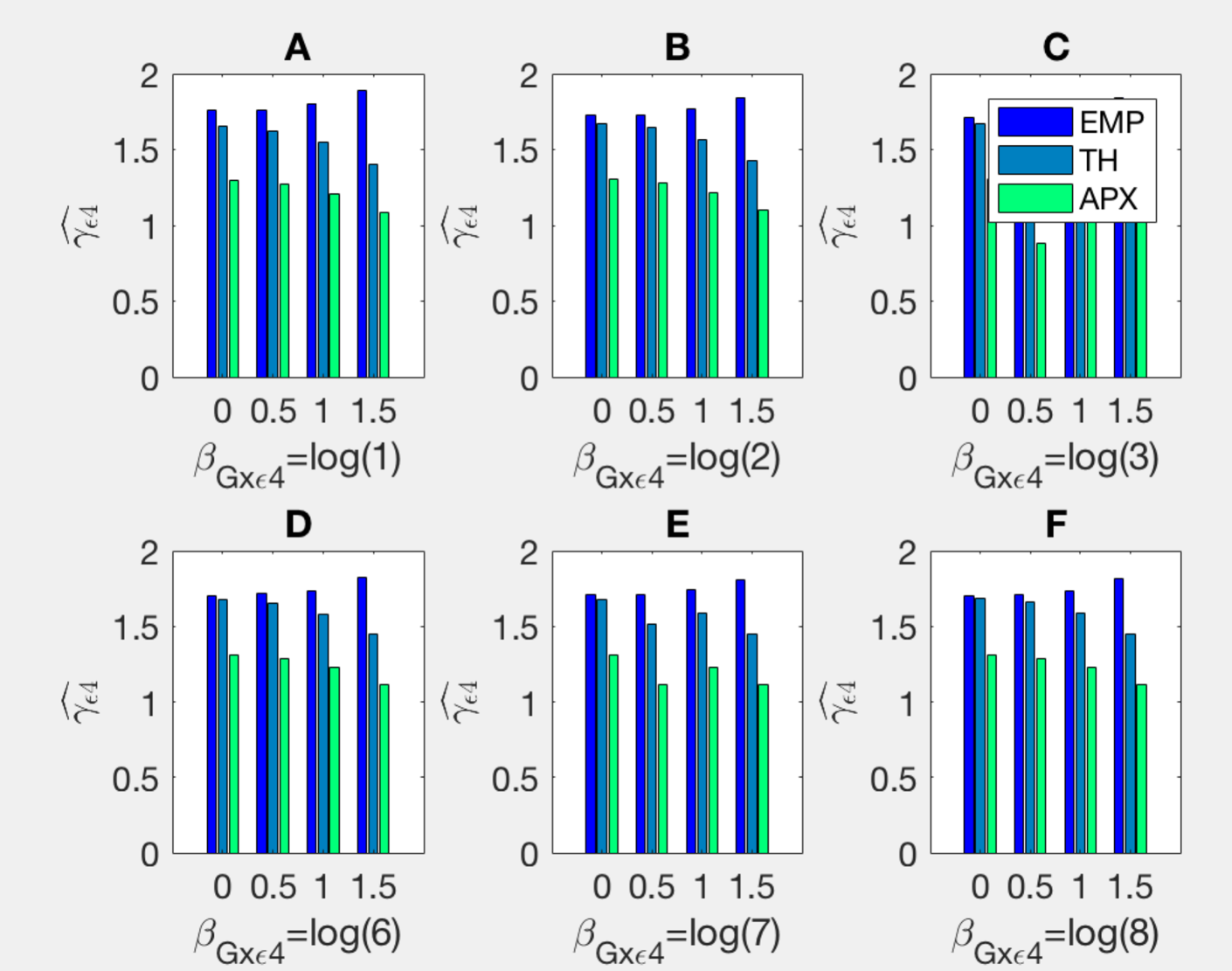


**Supplementary Figure 1B**: Estimates of $\gamma_{\varepsilon4}$ obtained empirically as an average across 500 simulated datasets (EMP); with 3,000 cases and 3,000 controls; using the theoretical formulae (A11)-(A15) as the difference in logs (TH); and using the approximation (A11)-(A15) (APX). Coefficients $\gamma$s are estimated based on misspecified model (3). The genetic variable is Bernoulli with frequency 0.1. The environmental variables $\varepsilon4,Z_{1}, Z_{2}$ are Bernoulli with frequencies 14%, 50%, and 52%, respectively. The *true* diagnosis is simulated based on the model (1) with coefficients $\beta_{0}=-1, \beta_{G}=0.41, \beta_{Z_{1}}=0, \beta_{Z_{2}}=-0.08, \beta_{\varepsilon4}=2.1$ and $\beta_{G\times\varepsilon4}=0.$The clinical-pathological diagnoses relationship is defined as $pr(D=1^{*}|D^{CL}=1,\varepsilon4-)=0.36$ and $pr(D=1^{*}|D^{CL}=1,\varepsilon4+)=0.06$.


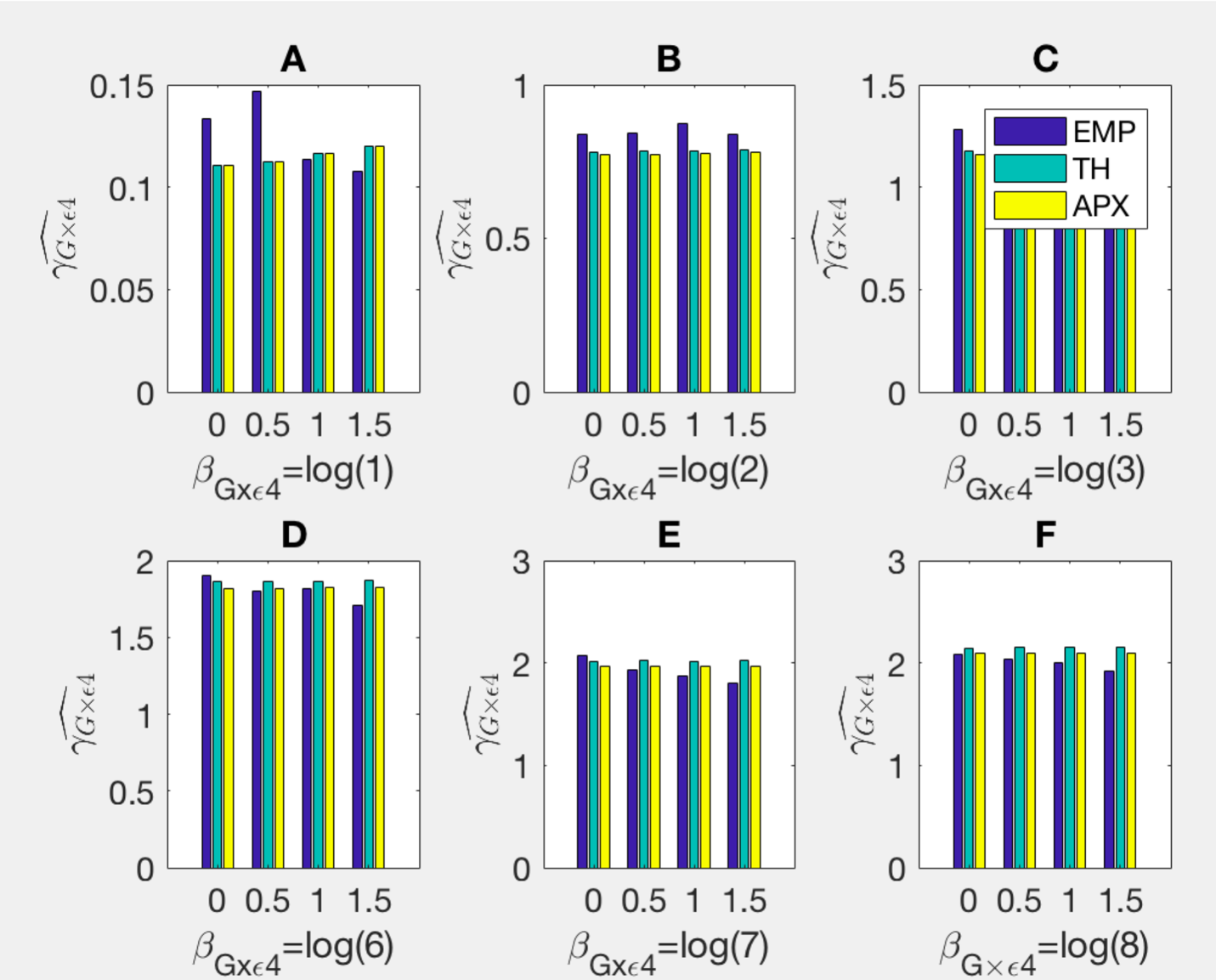


**Supplementary Figure 1C**: Estimates of $\gamma_{G\times\varepsilon4}$ obtained empirically as an average across 500 simulated datasets (EMP); with 3,000 cases and 3,000 controls; using the theoretical formulae (A11)-(A15) as the difference in logs (TH); and using the approximation (A11)-(A15) (APX). Coefficients $\gamma$s are estimated based on misspecified model (3). The genetic variable is Bernoulli with frequency 0.1. The environmental variables $\varepsilon4, Z_{1}, Z_{2}$ are Bernoulli with frequencies 14%, 50%, and 52%, respectively. The *true* diagnosis is simulated based on the model (1) with coefficients $\beta_{0}=-1, \beta_{G}=0.41, \beta_{Z_{1}}=0, \beta_{Z_{2}}=-0.08, \beta_{\varepsilon4}=2.1$ and $\beta_{G\times\varepsilon4}=0.$The clinical-pathological diagnoses relationship is defined as $pr(D=1^{*}|D^{CL}=1,\varepsilon4-)=0.36$ and $pr(D=1^{*}|D^{CL}=1,\varepsilon4+)=0.06$.


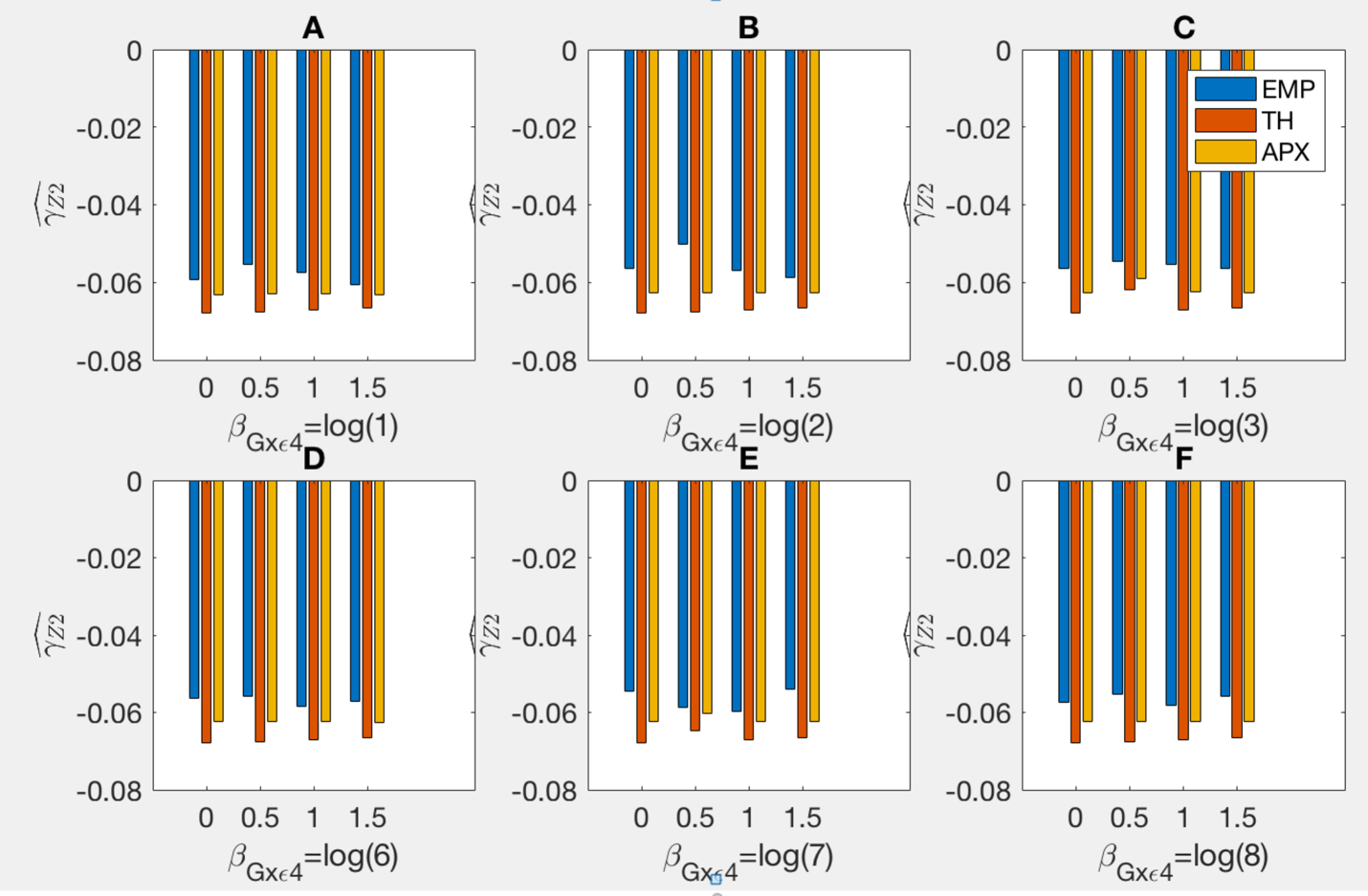


**Supplementary Figure 1D**: Estimates of $\gamma_{Z_{1}}$ obtained empirically as an average across 500 simulated datasets (EMP); with 3,000 cases and 3,000 controls; using the theoretical formulae (A11)-(A15) as the difference in logs (TH); and using the approximation (A11)-(A15) (APX). Coefficients $\gamma$s are estimated based on misspecified model (3). The genetic variable is Bernoulli with frequency 0.1. The environmental variables $\varepsilon4,Z_{1}, Z_{2}$ are Bernoulli with frequencies 14%, 50%, and 52%, respectively. The *true* diagnosis is simulated based on the model (1) with coefficients $\beta_{0}=-1, \beta_{G}=0.41, \beta_{Z_{1}}=0, \beta_{Z_{2}}=-0.08, \beta_{\varepsilon4}=2.1$ and $\beta_{G\times\varepsilon4}=0.$The clinical-pathological diagnoses relationship is defined as $pr(D=1^{*}|D^{CL}=1,\varepsilon4-)=0.36$ and $pr(D=1^{*}|D^{CL}=1,\varepsilon4+)=0.06$.

**
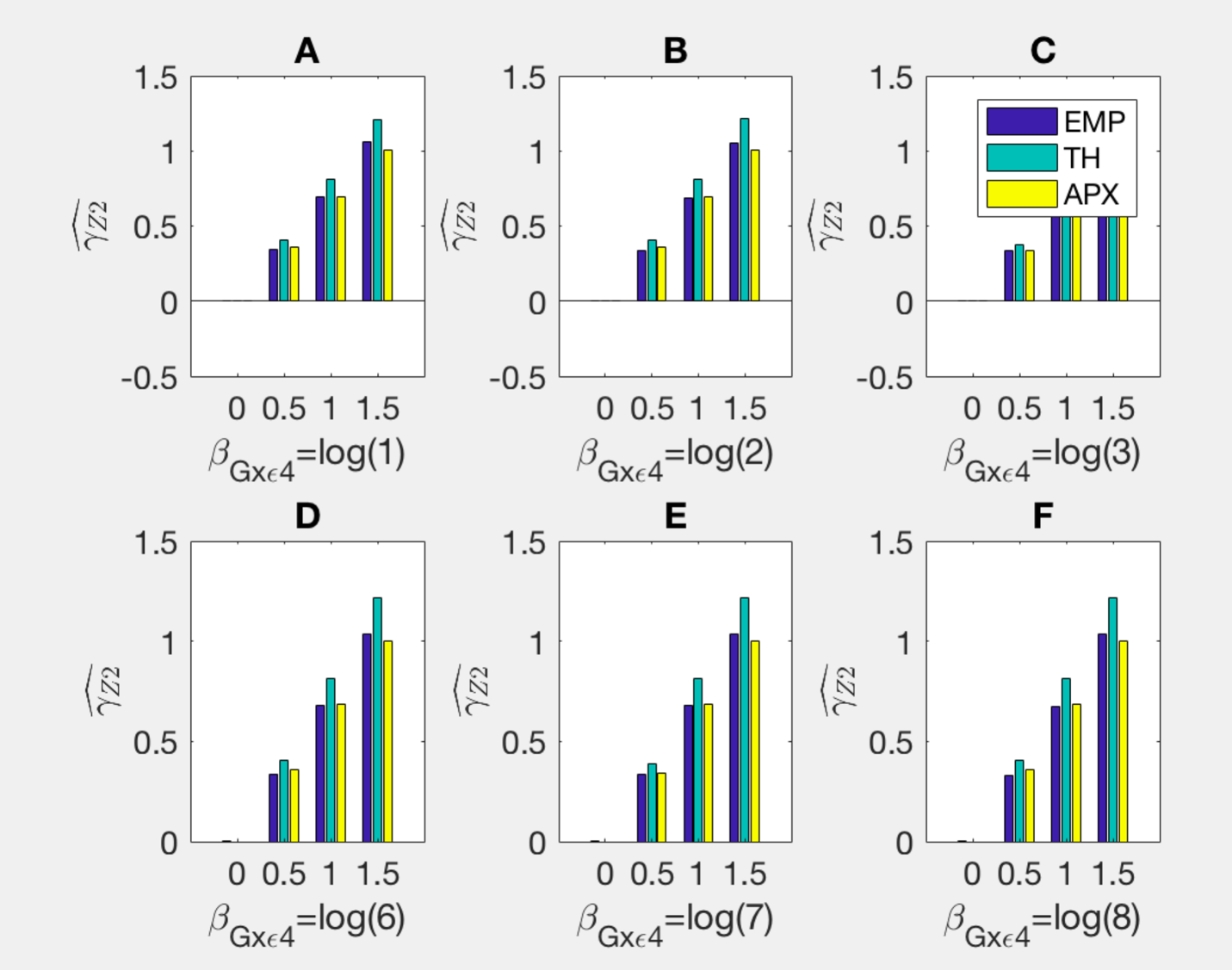
**

**Supplementary Figure 1E**: Estimates of $\gamma_{Z_{2}}$ obtained empirically as an average across 500 simulated datasets (EMP) with 3,000 cases and 3,000 controls; using the theoretical formulae (A11)-(A15) as the difference in logs (TH); and using the approximation (A11)-(A15) (APX). Coefficients $\gamma$s are estimated based on misspecified model (3). The genetic variable is Bernoulli with frequency 0.1. The environmental variables $\varepsilon4,Z_{1}, Z_{2}$ are Bernoulli with frequencies 14%, 50%, and 52%, respectively. The *true* diagnosis is simulated based on the model (1) with coefficients $\beta_{0}=-1, \beta_{G}=0.41, \beta_{Z_{1}}=0, \beta_{Z_{2}}=-0.08, \beta_{\varepsilon4}=2.1$ and $\beta_{G\times\varepsilon4}=0.$The clinical-pathological diagnoses relationship is defined as $pr(D=1^{*}|D^{CL}=1,\varepsilon4-)=0.36$ and $pr(D=1^{*}|D^{CL}=1,\varepsilon4+)=0.06$.


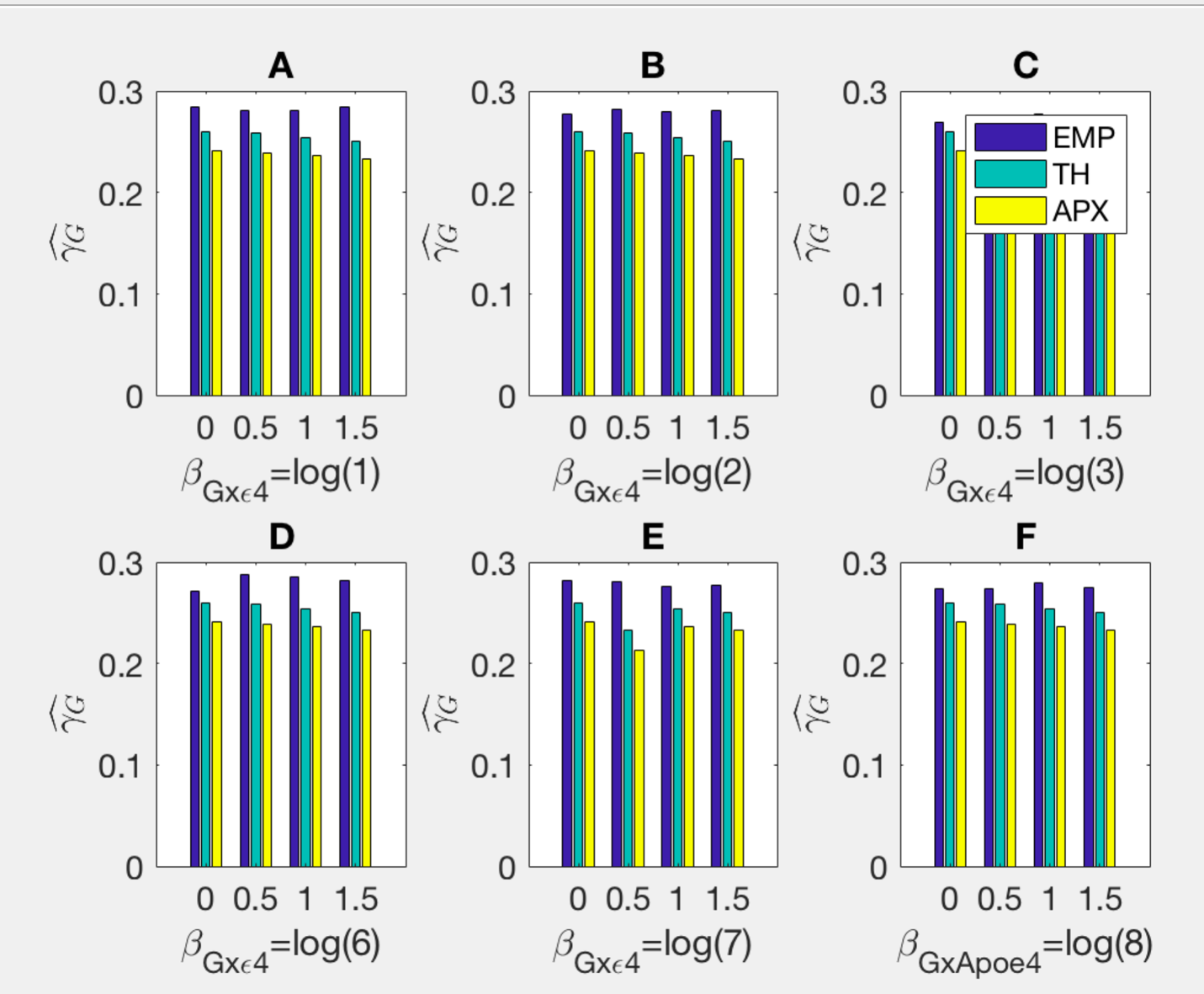


**Web Supplementary Figure 2A**: Estimates of $\gamma_{G}$ obtained empirically as an average across 500 simulated datasets (EMP) with 3,000 cases and 3,000 controls; using the theoretical formulae (A11)-(A15) (TH); and using the approximation (A11.1)-(A15.1) (APX). Coefficients $\gamma$s are estimated based on misspecified model (3).

The genetic variable is Bernoulli with frequency 0.1. The environmental variables $\varepsilon4,Z_{1}, Z_{2}$ are Bernoulli with frequencies 14%, 50%, and 52%, respectively. The *true* diagnosis is simulated based on the model (1) with coefficients $\beta_{0}=-1, \beta_{G}=0.41, \beta_{Z_{1}}=0, \beta_{Z_{2}}=-0.08, \beta_{\varepsilon4}=2.1$ and $\beta_{G\times\varepsilon4}=0.$The clinical-pathological diagnoses relationship is defined as $pr(D=1^{*}|D^{CL}=1,\varepsilon4-)=0.36$ and $pr(D=1^{*}|D^{CL}=1,\varepsilon4+)=0.06$, but in the theoretical derivations is underestimated to be $pr(D=1^{*}|D^{CL}=1,\varepsilon4-)=0.30$ and $pr(D=1^{*}|D^{CL}=1,\varepsilon4+)=0$.


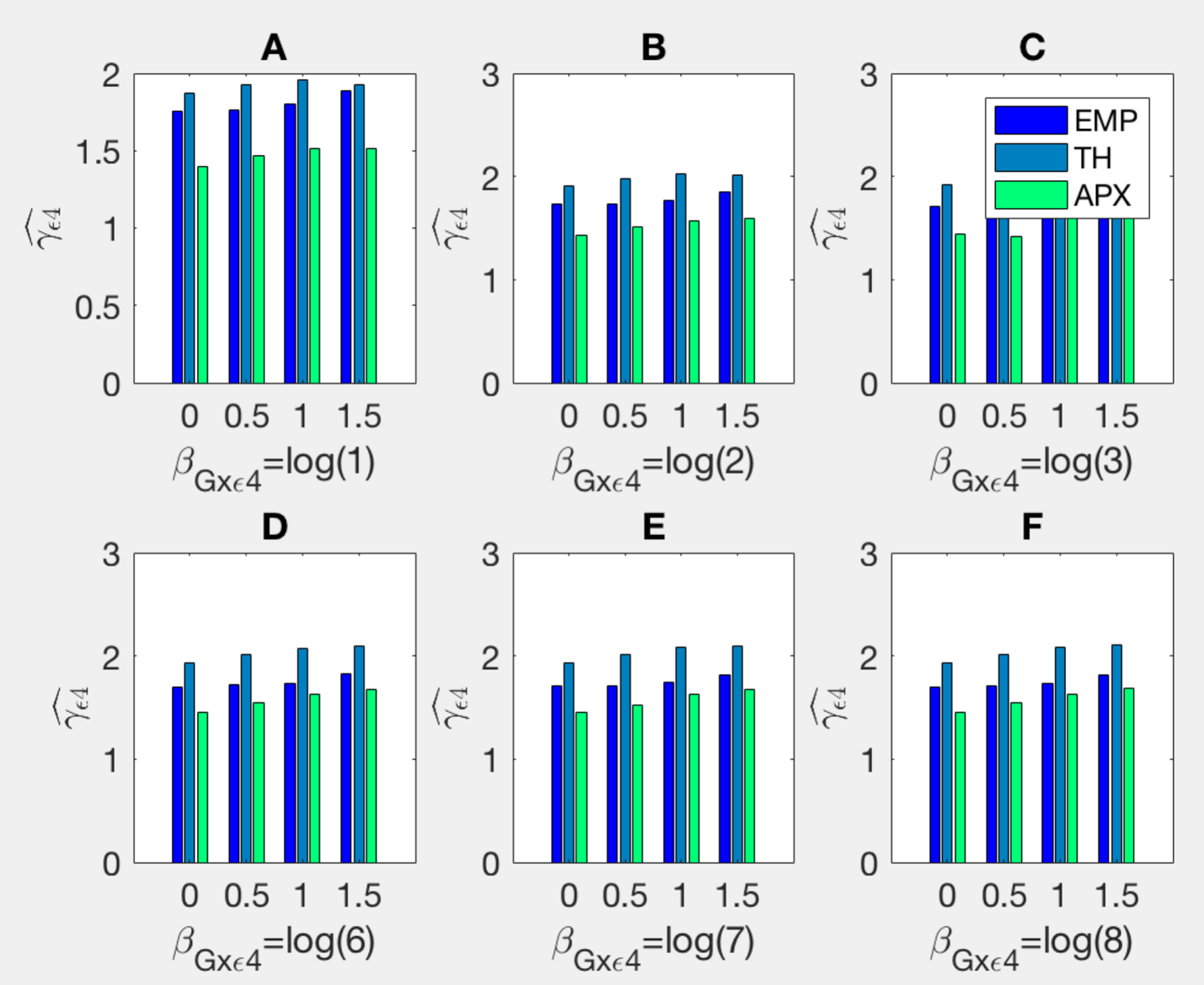


**Web Supplementary Figure 2B**: Estimates of $\gamma_{\varepsilon4}$ obtained empirically as an average across 500 simulated datasets (EMP); with 3,000 cases and 3,000 controls; using the theoretical formulae (A11)-(A15) as the difference in logs (TH); and using the approximation (A11)-(A15) (APX). Coefficients $\gamma$s are estimated based on misspecified model (3). The genetic variable is Bernoulli with frequency 0.1. The environmental variables $\varepsilon4,Z_{1}, Z_{2}$ are Bernoulli with frequencies 14%, 50%, and 52%, respectively. The *true* diagnosis is simulated based on the model (1) with coefficients $\beta_{0}=-1, \beta_{G}=0.41, \beta_{Z_{1}}=0, \beta_{Z_{2}}=-0.08, \beta_{\varepsilon4}=2.1$ and $\beta_{G\times\varepsilon4}=0.$The clinical-pathological diagnoses relationship is defined as $pr(D=1^{*}|D^{CL}=1,\varepsilon4-)=0.36$ and $pr(D=1^{*}|D^{CL}=1,\varepsilon4+)=0.06$, but in the theoretical derivations is underestimated to be $pr(D=1^{*}|D^{CL}=1,\varepsilon4-)=0.30$ and $pr(D=1^{*}|D^{CL}=1,\varepsilon4+)=0$.


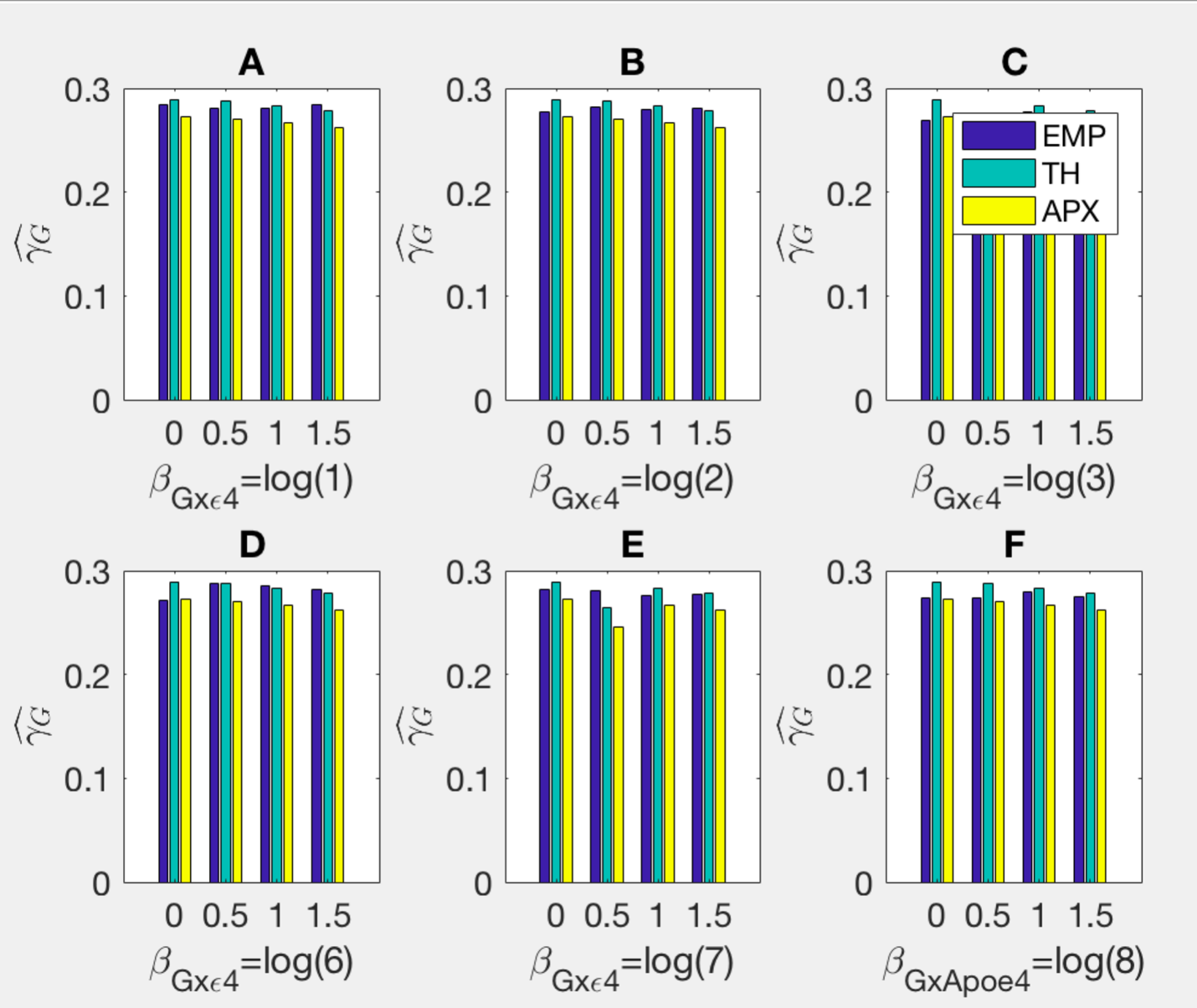


**Web Supplementary Figure 2C**: Estimates of $\gamma_{G\times\varepsilon4}$ obtained empirically as an average across 500 simulated datasets (EMP); with 3,000 cases and 3,000 controls; using the theoretical formulae (A11)-(A15) as the difference in logs (TH); and using the approximation (A11)-(A15) (APX). Coefficients $\gamma$s are estimated based on misspecified model (3). The genetic variable is Bernoulli with frequency 0.1. The environmental variables $\varepsilon4, Z_{1}, Z_{2}$ are Bernoulli with frequencies 14%, 50%, and 52%, respectively. The *true* diagnosis is simulated based on the model (1) with coefficients $\beta_{0}=-1, \beta_{G}=0.41, \beta_{Z_{1}}=0, \beta_{Z_{2}}=-0.08, \beta_{\varepsilon4}=2.1$ and $\beta_{G\times\varepsilon4}=0.$The clinical-pathological diagnoses relationship is defined as $pr(D=1^{*}|D^{CL}=1,\varepsilon4-)=0.36$ and $pr(D=1^{*}|D^{CL}=1,\varepsilon4+)=0.06$, but in the theoretical derivations is underestimated to be $pr(D=1^{*}|D^{CL}=1,\varepsilon4-)=0.30$ and $pr(D=1^{*}|D^{CL}=1,\varepsilon4+)=0$.


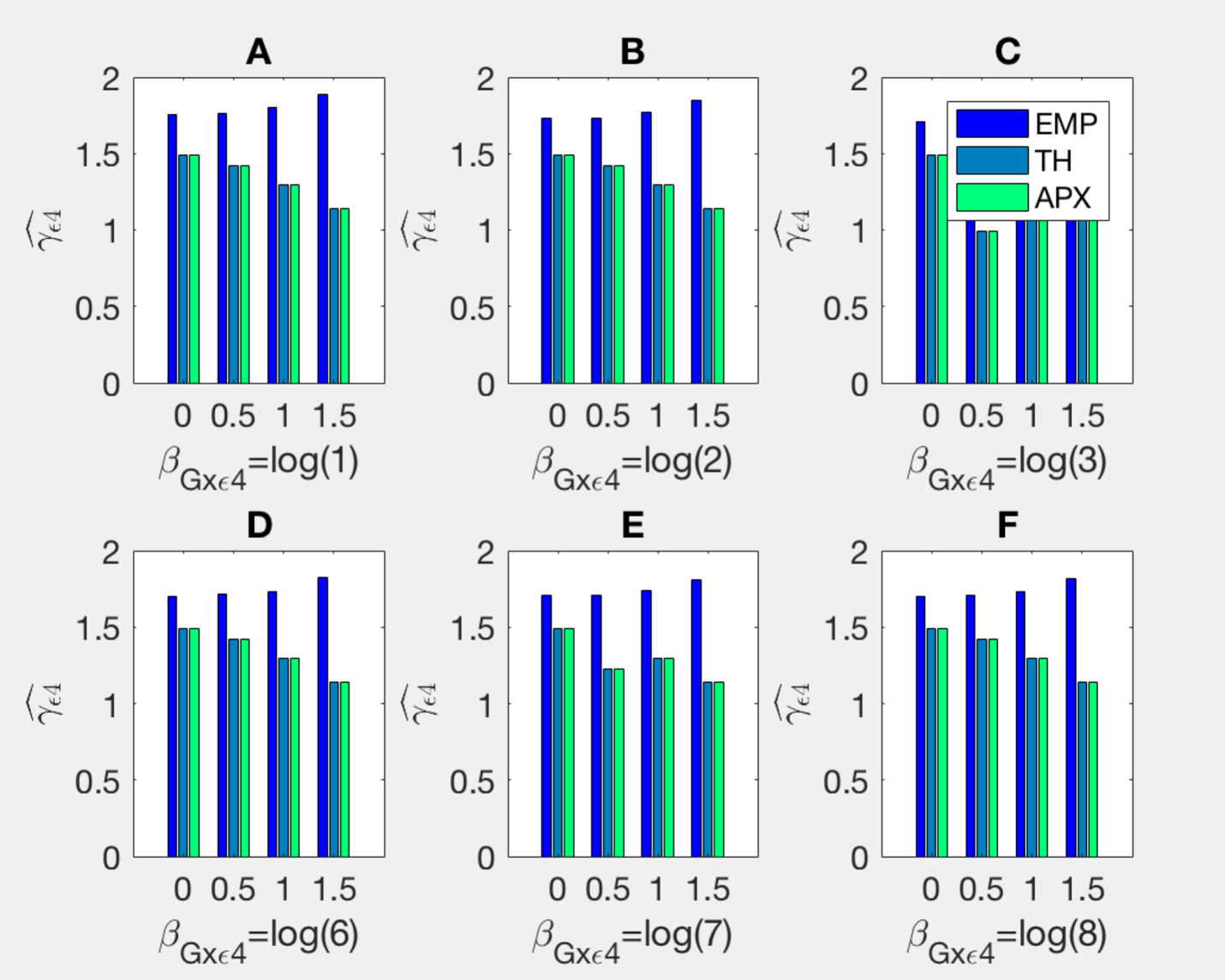


**Web Supplementary Figure 3A**: Estimates of $\gamma_{\varepsilon4}$ obtained empirically as an average across 500 simulated datasets (EMP); with 3,000 cases and 3,000 controls; using the theoretical formulae (A11)-(A15) as the difference in logs (TH); and using the approximation (A11)-(A15) (APX). Coefficients $\gamma$s are estimated based on misspecified model (3). The genetic variable is Bernoulli with frequency 0.1. The environmental variables $\varepsilon4,Z_{1}, Z_{2}$ are Bernoulli with frequencies 14%, 50%, and 52%, respectively. The *true* diagnosis is simulated based on the model (1) with coefficients $\beta_{0}=-1, \beta_{G}=0.41, \beta_{Z_{1}}=0, \beta_{Z_{2}}=-0.08, \beta_{\varepsilon4}=2.1$ and $\beta_{G\times\varepsilon4}=0.$The clinical-pathological diagnoses relationship is defined as $pr(D=1^{*}|D^{CL}=1,\varepsilon4-)=0.36$ and $pr(D=1^{*}|D^{CL}=1,\varepsilon4+)=0.06$, but in the theoretical derivations is overestimated to be $pr(D=1^{*}|D^{CL}=1,\varepsilon4-)=0.42$ and $pr(D=1^{*}|D^{CL}=1,\varepsilon4+)=0.12$.


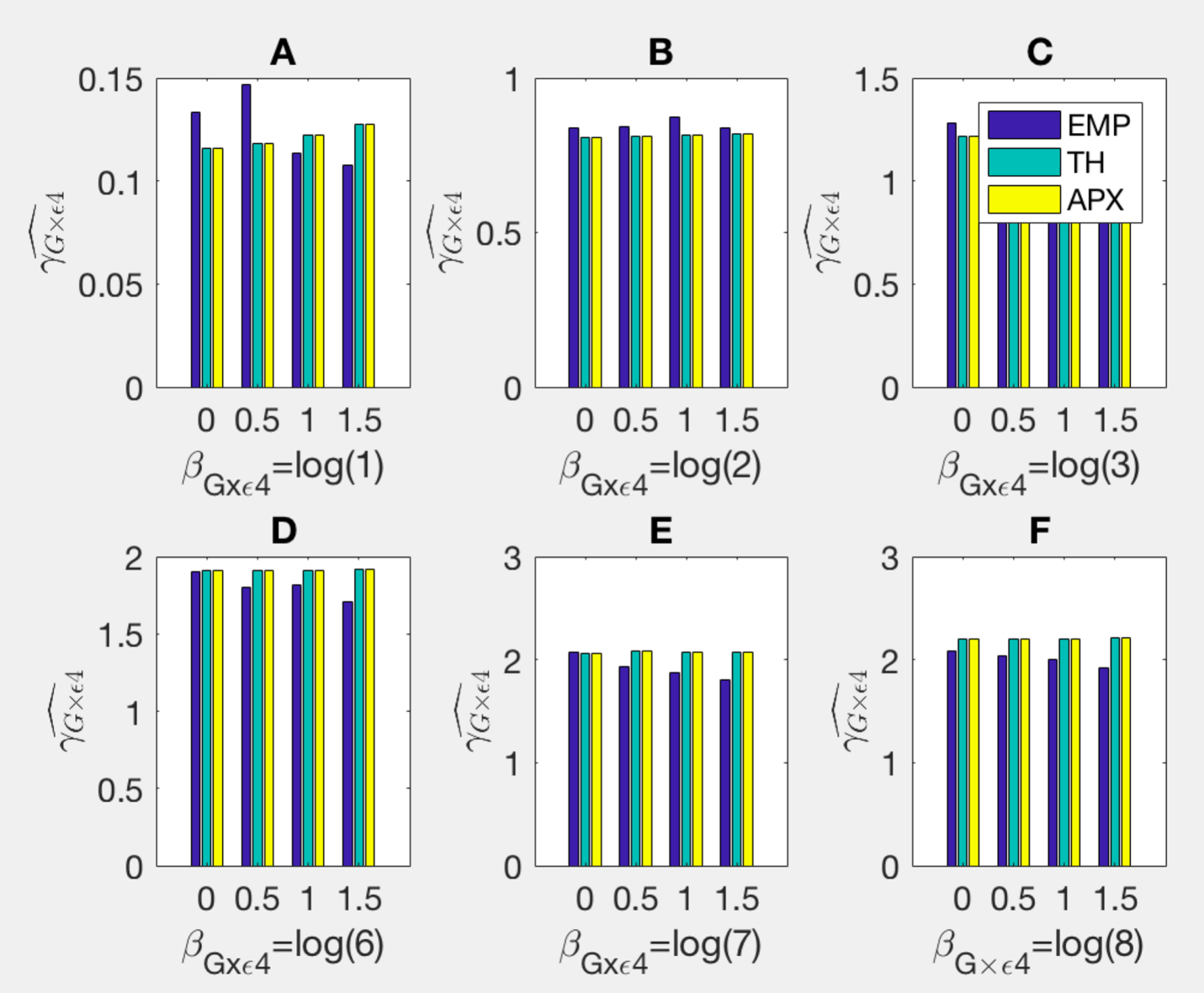


**Web Supplementary Figure 3B**: Estimates of $\gamma_{G\times\varepsilon4}$ obtained empirically as an average across 500 simulated datasets (EMP); with 3,000 cases and 3,000 controls; using the theoretical formulae (A11)-(A15) as the difference in logs (TH); and using the approximation (A11)-(A15) (APX). Coefficients $\gamma$s are estimated based on misspecified model (3). The genetic variable is Bernoulli with frequency 0.1. The environmental variables $\varepsilon4, Z_{1}, Z_{2}$ are Bernoulli with frequencies 14%, 50%, and 52%, respectively. The *true* diagnosis is simulated based on the model (1) with coefficients $\beta_{0}=-1, \beta_{G}=0.41, \beta_{Z_{1}}=0, \beta_{Z_{2}}=-0.08, \beta_{\varepsilon4}=2.1$ and $\beta_{G\times\varepsilon4}=0.$The clinical-pathological diagnoses relationship is defined as $pr(D=1^{*}|D^{CL}=1,\varepsilon4-)=0.36$ and $pr(D=1^{*}|D^{CL}=1,\varepsilon4+)=0.06$, but in the theoretical derivations is underestimated to be $pr(D=1^{*}|D^{CL}=1,\varepsilon4-)=0.30$ and $pr(D=1^{*}|D^{CL}=1,\varepsilon4+)=0$.


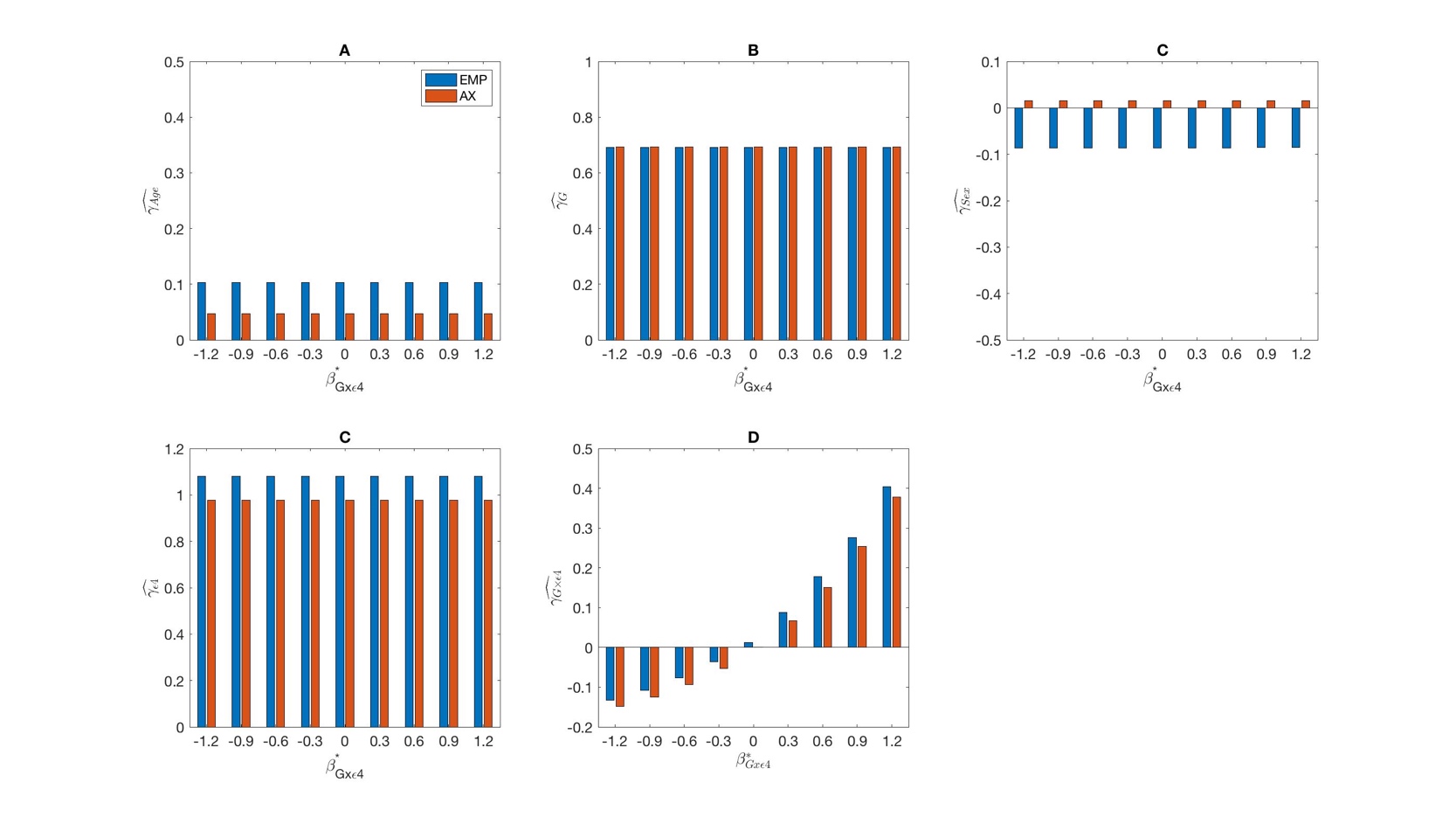


**Web Supplementary Figure 4A**: Empirical estimates (EMP) and approximation (AX) of parameter estimates when the data are simulated according to the risk model (2) while parameters are estimated based on the model (3). EMPs are the averages across 500 simulated datasets with 3,000 cases and 3,000 controls. Approximations are obtained using (A20)-(A23). We simulate the disease status *D*=1 vs. *D*=0 based on parameters$\beta_{0}=-1, \beta_{G}=-0.69, \beta_{Z_{1}(Age)}=0.10, {\beta_{Z_{2(Sex)}}=-0.083,\beta}_{\varepsilon4}=1.3, \beta_{G\times\varepsilon4}=0$; and we simulate *D*=$1^{*}$ vs. *D*=0 using $\beta_{0}^{*}=-1.7, \beta_{G}^{*}=0, , \beta_{\varepsilon4}^{*}=0.5, \beta_{G\times\varepsilon4}^{*}=-1.2, -0.9, -0.6, -0.3, 0, 0.3, 0.6, 0.9, 1.2$ with main effects of $Z_{1}$(Age) and $Z_{2}$(Sex) that are the same as for *D*=1 vs. *D*=0.


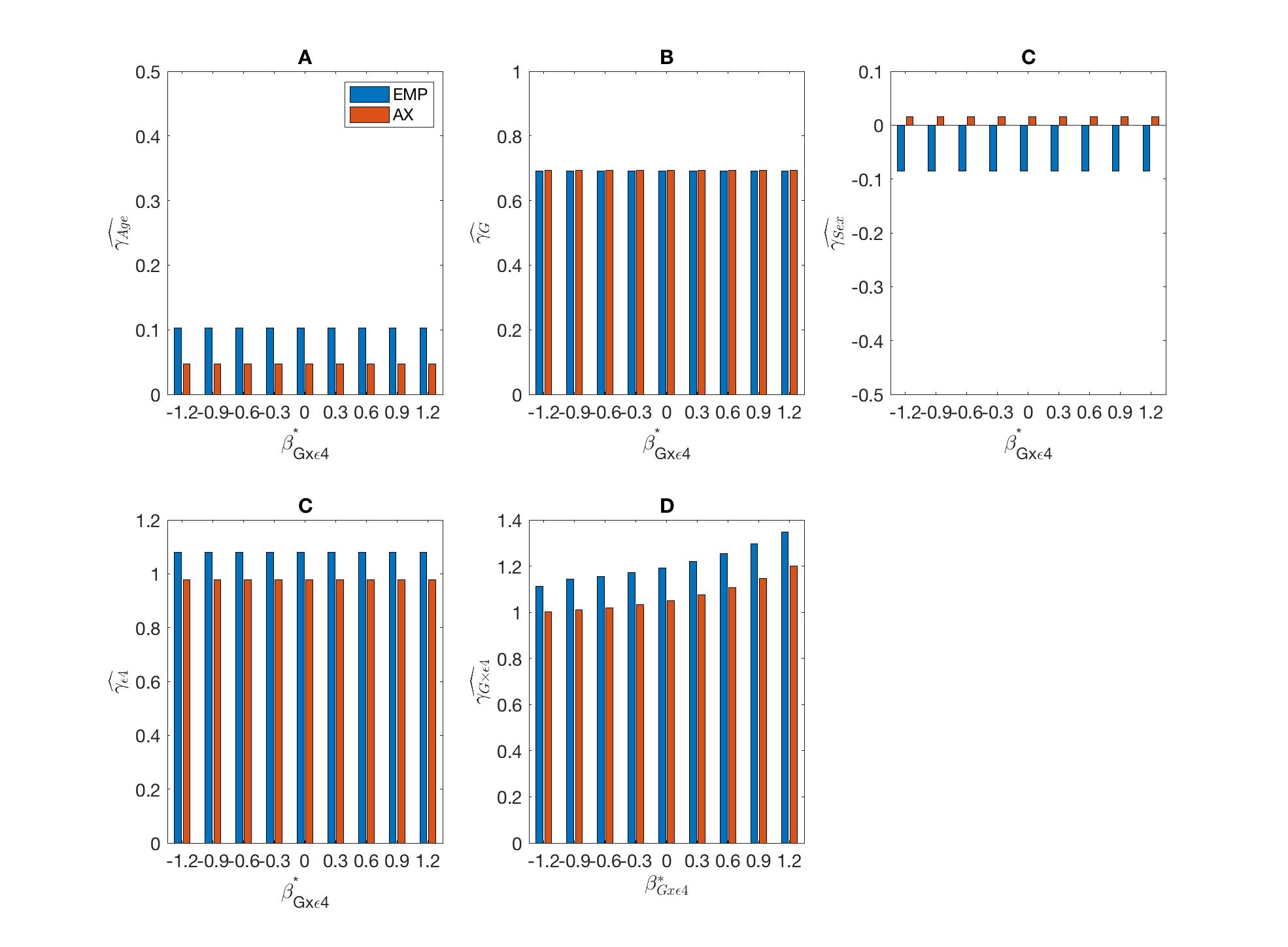


**Web Supplementary Figure 4B**: Empirical estimates (EMP) and approximation (AX) of parameter estimates when the data are simulated according to the risk model (2) while parameters are estimated based on the model (3). EMPs are the averages across 500 simulated datasets with 3,000 cases and 3,000 controls. Approximations are obtained using (A20)-(A23). We simulate the disease status *D*=1 vs. *D*=0 based on parameters$\beta_{0}=-1, \beta_{G}=-0.69, \beta_{Z_{1}\left( Age \right)}=0.10, {\beta_{Z_{2\left( Sex \right)}}=-0.083,\beta}_{\varepsilon4}=1.3, \beta_{G\times\varepsilon4}=-0.9$ and we simulate *D*=$1^{*}$ vs. *D*=0 using $\beta_{0}^{*}=-1.7, \beta_{G}^{*}=0, \beta_{\varepsilon4}^{*}=0.5, \beta_{G\times\varepsilon4}^{*}=-1.2, -0.9, -0.6, -0.3, 0, 0.3, 0.6, 0.9, 1.2$ with main effects of $Z_{1}$(Age) and $Z_{2}$(Sex) that are the same as for *D*=1 vs. *D*=0.
